# Histone lactylation-driven B7-H3 expression promotes tumor immune evasion

**DOI:** 10.7150/thno.105947

**Published:** 2025-01-13

**Authors:** Zhibo Ma, Jincui Yang, Wenlong Jia, Le Li, Yixin Li, Junjie Hu, Wei Luo, Ronghui Li, Dawei Ye, Peixiang Lan

**Affiliations:** 1 Institute of Organ Transplantation, Tongji Hospital, Tongji Medical College, Huazhong University of Science and Technology, 430030 Wuhan, China.; 2 Key Laboratory of Organ Transplantation, Ministry of Education; NHC Key Laboratory of Organ Transplantation; Key Laboratory of Organ Transplantation, Chinese Academy of Medical Sciences, 430030 Wuhan, People's Republic of China.; 3 Department of oncology, Tongji Hospital, Tongji Medical College, Huazhong University of Science and Technology, Wuhan, 430030, China.; 4 Institute of Urology, Tongji Hospital, Tongji Medical College, Huazhong University of Science and Technology, Wuhan, 430030, China.; 5 Department of neurosurgery, Affiliated Hospital of Shandong University of traditional Chinese Medicine, Weifang, 250100, China.; 6 Hepatic Surgery Center, Tongji Hospital, Tongji Medical College, Huazhong University of Science and Technology, 430030 Wuhan, China.

**Keywords:** B7-H3, Histone lactylation, H3K18la, Glycolysis, Immune evasion

## Abstract

**Rationale:** Tumor cells possess sophisticated strategies to circumvent immune detection, including the modulation of endogenous immune checkpoints, particularly those within the B7 family. Elucidating the mechanisms that govern the induction of B7 family molecules is crucial for the advancement of immunotherapy. Lysine lactylation (Kla), a newly identified epigenetic modification, is suggested may play a role in reshaping the tumor microenvironment and facilitating immune evasion.

**Methods:** We analyzed the glycolysis pathway's enrichment in patients with immune-evading tumors and assessed the impact of lactate treatment on the antitumor immunity of CD8^+^ T cells in the tumor microenvironment. We interrupted glycolysis using lactate dehydrogenase A (*LDHA*) knockdown and sodium oxamate, and evaluated its effects on CD8^+^ T cell cytotoxicity. Additionally, we investigated the correlation between B7-H3 expression and the glycolysis pathway, and explored the molecular mechanisms underlying lactate-induced B7-H3 expression.

**Results:** Our findings revealed that the glycolysis pathway was highly enriched in immune-evading tumors. Lactate treatment inhibited the antitumor immunity of CD8^+^ T cells, whereas interruption of glycolysis via LDHA knockdown or treatment with sodium oxamate augmented the cytotoxicity of CD8^+^ T cells, effectively counteracting tumor immune evasion. B7-H3 expression was found to be closely linked with the glycolysis pathway. Mechanistically, lactate-upregulated H3K18la directly bound to the B7-H3 promoter in conjunction with the transcription factor Creb1 and its co-activator Ep300, leading to increased B7-H3 expression and contributing to tumor progression by compromising the proportion and cytotoxicity of tumor-infiltrating CD8^+^ T cells. In mouse tumor bearing models, inhibiting glycolysis and B7-H3 expression suppressed tumor cell growth, activated tumor-infiltrating CD8^+^ T cells, and demonstrated potent anti-tumor efficacy. Furthermore, this approach enhanced the efficacy of anti-PD-1 treatment.

**Conclusions:** This study uncovers a novel mechanism by which lactate modulates the immune microenvironment through the glycolysis pathway and B7-H3 expression, providing new avenues for lactate metabolism-targeted tumor immunotherapy.

## Introduction

The emergence of immunotherapy has dramatically revolutionized the treatment landscape for melanoma and multiple advanced solid malignancies 1. Immune checkpoint inhibitors (ICIs), including antibodies targeting the programmed cell death 1 (PD-1)-PD-1 ligand 1 (PD-L1) axis (such as nivolumab and pembrolizumab) and cytotoxic T lymphocyte antigen 4 (CTLA4) (like ipilimumab), have gained approval from the US Food and Drug Administration (FDA) for the treatment of various cancers, which conferring a notable survival advantage to numerous patients 2. Despite their remarkable success, it should be noted that the clinical response to anti-PD-1 therapy is limited, with a significant proportion of patients demonstrating either no response or acquiring resistance following an initial positive outcome 3, 4. Furthermore, beyond the currently actionable checkpoints mentioned above, other immune checkpoint molecules expressed by tumor cells may also impede the desired antitumor effects in the tumor microenvironment (TME).

B7-H3, also known as CD276, is a transmembrane protein belonging to the B7 family 5. The B7 family encompasses various molecules, also including the CTLA-4 ligands B7-1 (CD80) and B7-2 (CD86), B7-H1 (PD-L1), B7-DC (PD-L2), B7-H2 (ICOS-L) and B7-H4. B7-H3 shared 88% amino acid identity between humans and mice, and there is a 28% amino acid identity with PD-L1 5. Initially characterized as a costimulatory molecule, B7-H3 is now recognized for its involvement in immunosuppression and tumor progression, while the B7-H3 receptor has not been clearly elucidated. Specifically, it predominantly exerts its role in suppressing the anti-tumor immune responses, primarily mediated by T cells and NK cells 6. While B7-H3 is normally expressed at low levels in healthy tissues 7, it displays abnormal overexpression in various carcinomas such as melanoma 8, prostate cancer 9, liver cancer 10, non-small-cell lung cancer 11, and ovarian cancer 12. Notably, increased expression of B7-H3 is relevant to the enhanced risks of clinical recurrence, therapy resistance, immune evasion, and poorer prognosis 6. Indeed, B7-H3 expression reduces CD8^+^ T cell activation, as well as cytotoxic cytokine release 13. In addition, B7-H3 silencing and ablation promotes T cells activation and accelerates auto-immune encephalomyelitis 14. In graft-versus-host-disease, B7-H3 deletion resulted in uncontrolled Th1 responses 15. Tumor cells with B7-H3 exhibit a dominant role in suppressing antitumor immunity, and is insensitive to PD-1 blockade therapy 12. Despite these significant findings, the exact mechanism of B7-H3 in remodeling the TME and the mechanisms regulating its expression remain incompletely explored.

Post-translational modifications of histones critically regulate gene expression and contribute to tumor initiation and progression. Interestingly, Zhang *et al.* discovered a novel epigenetic modification, called histone lactylation 16. Lactate is necessary for histone lactylation, inducing the expression of repair genes during M1 macrophage polarization, and favors cancer initiation and progression 16. The Warburg effect, characterized by enhanced glycolysis and lactate accumulation, represents a significant hallmark of cancer. Consequently, it is plausible that histone lactylation may also exhibit aberrant patterns in tumors, warranting investigation into its potential role in tumorigenesis. Histone H3 lysine 18 lactylation (H3K18la) promotes the expression of POM121, which facilitates the nuclear import of MYC to enhance the expression of PD-L1 17. Furthermore, researches have demonstrated that histone lactylation accelerates oncogenesis in ocular melanoma by upregulating the expression of YTHDF2, which leads to the degradation of m6A-modified PER1 and TP53 mRNAs 18. In colorectal carcinoma, histone lactylation has been implicated in driving METTL3-mediated RNA m6A modification, promoting immune suppression in tumor-infiltrating myeloid cells and mediating tumor immune evasion 19. As research advances, further investigations have substantiated the involvement of lactylation modification in tumor development 20, ischemic heart 21, macrophage polarization 16, 22, and nervous system regulation 23.

In this study, we made a significant finding demonstrating that the glycolysis pathway was highly enriched in patients with immune evasion. Lactate induces the upregulation of the B7-H3 gene via histone lactylation on the surface of tumor cells. This upregulated expression of B7-H3 subsequently exerts an inhibitory effect on infiltration ratio and cytotoxicity of CD8^+^ T cells infiltrating the TME, thereby promoting tumor progression. Notably, we observed a synergistic effect when combining a lactate dehydrogenase (LDH) inhibitor with a PD-1 inhibitor, leading to a substantial inhibition of tumor progression. These findings offer a novel avenue for clinical tumor treatment by targeting lactate metabolism in combination with ICIs.

## Material and methods

### Patients and samples

Human hepatocellular carcinoma (HCC) tissues and adjacent normal tissues were recruited from the Hepatobiliary Center of Tongji Hospital, Huazhong University of Science and Technology (HUST), Wuhan, China. Written informed consent was meticulously obtained from each patient involved in the study.

### Mice and cell lines

Male C57BL/6 wild-type mice (6-8 weeks old) were purchased from Beijing Vital River Laboratory Animal Technology Co. Ltd (Beijing, China) and housed in the Laboratory Animal Center of Scientific Research Building, Tongji Hospital, Tongji Medical College, Huazhong University of Science and Technology. The experimental protocol was approved by the Ethics Committee of Tongji Hospital, Tongji Medical College, Huazhong University of Science and Technology. All animal handling procedures adhered to the relevant guidelines and regulations set forth by the Chinese Council on Animal Care. Murine melanoma B16 and Hepa1-6 cell lines were purchased from ATCC. Cells were cultured with DMEM/1640 medium supplemented with 10% fetal bovine serum and 100 U/mL penicillin-streptomycin.

### Mouse tumor models

For subcutaneous tumor inoculation, lactate (LA) treated, B7-H3 knockout (KO), B7-H3 KO + LA, wild-type (WT) B16 or Hepa1-6 cells, stably expressing empty vector (pCMV-flag) and overexpression B7-H3 (pCMV-flag B7-H3) B16/Hepa1-6 cells, resuspended in of PBS to make a concentration of 1×10^7^ cells/mL suspension. Subsequently, 1×10^6^ cells (in 100 μL of PBS) were inoculated subcutaneously into the flanks of male C57BL/6J mice, aged six to eight weeks.

For sodium lactate treatment, daily doses of PBS or 1 g kg^-1^ sodium lactate were administrated intraperitoneally seven days before the inoculation of B16/Hepa1-6 cells. Injections were continued throughout the experiments.

For sodium oxamate and PD-1 antibody treatment experiments, mice were randomly assigned and were intraperitoneally administered rat IgG1 isotype control antibody injections (BioxCell, BE0088), anti-PD-1 (200 μg/mouse, BioxCell, BE0146) antibody, LDH inhibitor sodium oxamate (300 mg/kg, Topscience, T19831). The anti-PD-1 antibody treatment was initiated from day 7 and repeated every 3 days for a total of 3 treatments, while sodium oxamate was administered every two days. Mice were euthanized 24 hours following the final treatment. To deplete CD8^+^ T cells *in vivo*, mice received an initial intraperitoneal injection of InVivoMAb anti-CD8 monoclonal antibody (200 μg/mouse) two days before B16 cells were inoculated and then twice a week. Tumor growth was assessed starting from day 7 after inoculation, and tumor volumes were recorded every 2 days using the formula: tumor volume = 0.5 × (small diameter)^2^ × (large diameter). After a 14-day period, the mice were euthanized for further phenotypic analyses.

### Isolation of immune-infiltrating cells from tumor tissues

Tumor-bearing mice were sacrificed and tumor tissues were isolated on post-inoculation day 14. The tumor tissue was homogenized with a syringe, washed with PBS, and pressed through a 70 µm filter to achieve single-cell suspensions. After collection, the cells were thoroughly washed with PBS, and tumor-infiltrating monocytes were subsequently isolated using density gradient centrifugation with a 40% Percoll solution (Biosharp). Erythrocytes were lysed using red cell lysate (Servicebio) on ice for 5-8 min and the reaction was stopped with ice-cold PBS. Centrifuged at 1800 rpm for 5 min, the collected cells were subjected to flow cytometry analysis.

### Quantitative RT-PCR

For quantitative RT-PCR, total RNA from culture cells was extracted using a total RNA rapid extraction kit (Fastagen, China), and reverse-transcribed into complementary DNA using PrimeScript PT Master Mix (TAKARA, Japan). Quantitative real-time PCR (qPCR) was using SYBR qPCR Master Mix (Vazyme, China) performed on the Applied Biosystems StepOne System (Thermo Fisher Scientific). Gene expression levels were determined using the comparative Ct method and normalized to endogenous β-actin expression. The primer sequences utilized in this study are provided below:

B7-1-F: 5'-ACCCCCAACATAACTGAGTCT-3', B7-1-R: 5'-TTCCAACCAAGAGAAGCGAGG-3'; B7-2-F: 5'-TGTTTCCGTGGAGACGCAAG-3', B7-2-R: 5'- TTGAGCCTTTGTAAATGGGCA-3', B7-H1-F: 5'-GCTCCAAAGGACTTGTACGTG-3', B7-H1-R: 5'-TGATCTGAAGGGCAGCATTTC-3'; B7-H2-F: 5'-TAAAGTGTCCCTGTTTTGTGTCC-3', B7-H2-R: 5'-ATTGCACCGACTTCAGTCTCT-3'; B7-H3-F: 5'-GGCTGTATTCCCCTCCATCG-3'; B7-H3-R: 5'-CCAGTTGGTAACAATGCCATGT-3'; B7-H4-F: 5'-CTTTGGCATTTCAGGCAAGCA-3', B7-H4-R: 5'-TGATGTCAGGTTCAAAAGTGCAG-3'; B7-H5-F: 5'-GGAACCCTGCTCCTTGCTATT-3'; B7-H5-R: 5'-TTGTAGATGGTCACATCGTGC-3'; Galectin9-F: 5'-ATGCCCTTTGAGCTTTGCTTC-3'; Galectin9-R: 5'-AACTGGACTGGCTGAGAGAAC-3'; LDHA-F: 5'-TGTCTCCAGCAAAGACTACTGT-3'; LDHA-R: 5'-GACTGTACTTGACAATGTTGG GA-3'; β-actin-F: 5'-ATGCTTCGAGGATGGGGTG-3'; β-actin-R: 5'-CCAGGCTCTGGGGAAAAGG-3'.

### Cut-Tag qPCR

The Cut-Tag qPCR experiments were conducted using Hyperactive Universal CUT&Tag Assay Kit from Vazyme (TD904), and experiments were conducted according to the instructions. The primer sequences utilized in this study are provided below:

B7-H1-F: 5'-TCTGGAAAGGCGTGTTGGGAAG-3', B7-H1-R: 5'-CTCCGAGGCTGACAGGTATTGC-3'; B7-H2-F: 5'-TCTGTCTGTCTGTCTGTCT-3', B7-H2-R: 5'- TTCTGCCTGCTATGAACC-3'; B7-H3-F: 5'-CCACATCAACCACTAATCAAG-3'; B7-H3-R: 5'-GCACACTTATTCCTCTATTCTC-3'; DNA Spike in-F: 5'-GCCTTCTTCCCATTTCTGATCC-3', DNA Spike in-R: 5'-CACGAATCAGCGGTAAAGGT-3'.

### Cell viability assay

The proliferation of B16 cells under diverse experimental conditions was utilized a Cell Counting Kit-8 kit (CCK-8, Servicebio). These conditions included cells transduced with an empty vector, cells overexpressing B7-H3, cells with B7-H3 knocked out, and cells treated with L-lactate (LA) at a concentration of 20 mM. B16 cells were seeded in 96-well plates with an approximate density of 8000 cells per well. The plates were incubated in a 5% CO_2_ incubator, maintaining a temperature of 37 °C for varying durations of 24 hours, 48 hours, 72 hours, and 96 hours. After the incubation period, the cells were subjected to a 2-hour incubation with the CCK-8. This reagent facilitates the assessment of cell proliferation by measuring cell viability. The absorbance of the samples was then quantified at a wavelength of 450 nm, through which the proliferation of the cells could be determined.

### Flow cytometry

Single-cell suspensions underwent staining with various fluorochrome-conjugated antibodies at 4 °C for 30 min, and then subjected to analysis using the highly efficient BD FACSCelesta cell analyzer (BD Biosciences). For intracellular cytokine staining, including IFN-γ and TNF-α, cellular stimulation was achieved by subjecting the cells to a cell stimulation cocktail containing the protein transport inhibitor (eBioscience, 00-4975-93) for 4.5 hours at 37 °C. Next, the staining of membrane molecules was conducted utilizing specific antibodies targeted against the surface protein. This was followed by fixation and permeation procedures using BD Fix/Perm buffer kits, subsequently facilitating the staining process with antibodies that selectively target intracellular antigens. Intracellular staining of Granzyme B and perforin were performed after fixation and permeation without cell stimulation. The resulting data obtained from the flow cytometric analysis were meticulously analyzed using FlowJo v10.5.3 (TreeStar). A list of all antibodies is provided in Table S1.

### Western blotting

Cellular lysis was achieved by employing the RIPA lysis buffer supplemented with a protease inhibitor cocktail (Sigma, P8340). The BCA protein assay kit (Beyotime, China) was utilized to determine protein concentration, and denaturation was performed at 95 °C for 5 min. The separation of proteins was accomplished via the use of 10% SDS-PAGE and transferred to PVDF membranes. The membranes were subjected to a 2-hour blocking process at 37 °C, using a solution containing 5% nonfat milk. The primary antibodies were then incubated overnight at a temperature of 4 °C, after which the membranes were washed and incubated with secondary antibodies on the subsequent day. Finally, the blots were rendered visible employing an enhanced chemiluminescence (Biosharp). Primary antibodies used in this study were Pan anti-Kla (PTM-1401, PTM Bio), anti-H3K18la (PTM-1406, PTM Bio), anti-H3K9la (PTM-1419RM, PTM Bio), anti-H3K14la (PTM-1414RM, PTM Bio), anti-H3K27la (PTM-1428, PTM Bio), anti-H4K5la (PTM-1407, PTM Bio), anti-H4K8la (PTM-1415, PTM Bio), anti-H4K16la (PTM-1417RM, PTM Bio), anti-B7-H1 (Cat no : 66248-1-Ig, Proteintech), anti-B7-H2 (Cat no : 18251-1-AP, Proteintech), anti-B7-H3 (Cat no: ab134161, Abcam), anti-Creb1 (Cat no : 12208-1-AP, Proteintech), anti-Ep300 (Cat no : 86377S, CST).

### Immunoprecipitation

A total of 1x10^7^ tumor cells were collected and lysed in NETN lysis buffer. The cell lysate was then incubated with anti-Ep300 antibody or anti-H3K18la or anti-Creb1or control antibody at 4 °C for 6 hours. Next, the lysate was treated with Magna ChIP™ Protein A + G Magnetic Beads (Merck) overnight. The beads were collected through centrifugation and washed 4 times with IP lysis buffer. Finally, the proteins were resolved in PBS and subjected to analysis using immunoblotting.

### Immunofluorescence staining

Tissues were fixed in 4% formaldehyde and dehydrated using xylene, absolute ethyl alcohol, and 75% alcohol. Subsequently, they were embedded in paraffin wax. Sections four microns thick were then prepared for analysis. Staining of tissues were performed with corresponding antibodies.

### Immunohistochemistry (IHC)

Slides were rehydrated in xylene and ethanol after being deparaffinized for IHC. Following a 30-minute incubation period with 0.3% hydrogen peroxide, antigen retrieval was carried out using citrate buffer for 15 minutes at a temperature below boiling point, and the reaction was blocked for 60 minutes with 5% bovine serum albumin (BSA) and overnight at 4 °C with primary antibodies (specified in the CTAT_table). After that, the slides were then incubated for one hour at 37 °C with HRP-conjugated secondary antibodies. After that, the sections were incubated for color development using a 3, 3'-diaminobenzidine tetrahydrochloride kit (Gene Tech, Shanghai, China) and for nuclear counterstaining using hematoxylin. The program Case-Viewer (3DHISTECH, Budapest, Hungary) or a regular microscope (Olympus, Tokyo, Japan) were used to obtain the images.

### LC‒MS/MS analysis

LC‒MS/MS experiments were performed on a Q Exactive HF-X mass spectrometer that was coupled to an Easy nLC1200 (Thermo Scientific). The peptide was first loaded onto a trap column (100 μm*20 mm, 5 μm, C18, Dr. Maisch GmbH, Ammerbuch, Germany) in buffer A (0.1% formic acid in water). Reverse-phase high-performance liquid chromatography (RP-HPLC) separation was performed using a self-packed column (75 μm × 150 mm; 3 μm ReproSil-Pur C18 beads, 120 Å, Dr. Maisch GmbH, Ammerbuch, Germany) at a flow rate of 300 nL/min. The RP-HPLC mobile phase A was 0.1% formic acid in water, and B was 0.1% formic acid in 95% acetonitrile. The gradient was set as follows: 2%-4% buffer B from 0 min to 2 min, 4% to 30% buffer B from 2 min to 47 min, 30% to 45% buffer B from 47 min to 52 min, 45% to 90% buffer B from 52 min to 54 min, and 90% buffer B from 60 min. MS data were acquired using a data-dependent top 20 method that dynamically choosing the most abundant precursor ions from the survey scan (350-1800 m/z) for HCD fragmentation. A lock mass of 445.120025 Da was used as an internal standard for mass calibration. Full MS scans were acquired at a resolution of 60,000 at m/z 200 and 15,000 at m/z 200 for the MS/MS scan. The maximum injection time was set to 50 ms for MS and 25 ms for MS/MS. The normalized collision energy was 28, and the isolation window was set to 1.6 Th. The dynamic exclusion duration was 30 s.

### Generation of B7-H3 KO cells using CRISPR/Cas9

Using Benchling CRISPR sgRNA design tools (https://benchling.com/crispr) to design B7-H3 sgRNA sequence of gene targeting (B7-H3 KO-sgRNA: GCGCGTCCGAGTAACCGACG). The sgRNA oligonucleotide was integrated into the pSpCas9 (BB)-2A-Puro (PX459) plasmid subsequent to performing Bbs I digestion. Afterwards, the recombinant vector was introduced into B16 and Hepa1-6 cells using Neofect™ DNA transfection reagent (Neofect). Following a 48-hour period post-transfection, puromycin (1 μg/mL) was employed to facilitate the identification of colonies. Single cells were spread in 96-well plates using limited dilution, and individual colonies were selected microscopically one week later. Half of the cells were removed when the cells were fully grown, half were sent to sequencing to verify the knockout efficiency, and the remaining half was continued to culture. For the cells that were sequenced successfully, the depletion of B7-H3 protein in the clones was finally established through analysis using flow cytometry.

### Plasmid construction and lentiviral infection

The genes of B7-H3 coding sequences were exponentially amplified using the polymerase chain reaction (PCR) method, and subsequently integrated into pCMV-Flag vectors in accordance with the specific indications. The B7-H3 overexpression recombinant vector was verified by DNA sequencing. The plasmids were introduced into B16/Hepa1-6 cells using the Neofect™ DNA transfection reagent (Neofect). B16/Hepa1-6 cells were transduced with lentiviral particles encoding B7-H3, and selected with puromycin (P8230, Solarbio) for one mouth to generate stable cell lines. The overexpression efficiency was verified by qPCR and flow cytometry. Primers consist of the following: F: 5'-GGATCGGGTTTAAACGGATCCGCCACCATGCTTCGAGGATGGGGTGG-3', R: 5'-ATCGGGCCCTCTAGACTCGAGTCAAGCAATTTCTTGTCCGT-3'.

To construct a plasmid with knocked-down LDHA, we employed a pCDH-U6-shRNA-EF1-Puro empty vector as the control group and utilized AgeI and EcoRI endonucleases for vector digestion. Subsequently, we ligated to primers of LDHAsh1 sequences using T4 DNA ligase. Primers consist of the following: PCDH-LDHAsh1-F: 5'-CCGGCGTGAACATCTTCAAGTTCATCTCGAGATGAACTTGAAGATGTTCACGTTTTTG-3', PCDH-LDHAsh1-R: 5'-AATTCAAAAACGTGAACATCTTCAAGTTCATCTCGAGATGAACTTGAAGATGTTCACG-3'.

### Chromatin immunoprecipitation-sequencing (ChIP-seq)

Chromatin immunoprecipitation assays were conducted in accordance with established methods 24, as previously described by Wuhan IGENEBOOK Biotechnology Co, Ltd (http://www.igenebook.com). B16 cells were treated with LA (20 mM) for 3 days, and PBS was added to the control group. Briefly, 1×10^7^ cells per sample underwent two rounds of cold PBS buffer washing before being subjected to cross-linking with 1% formaldehyde for a duration of 10 minutes at room temperature. Following that, the cross-linking process was halted by introducing glycine at a final concentration of 125 mmol/L. The samples were then lysed using a buffer containing 1% SDS, 10 mM EDTA, 50 mM Tris-HCl (pH 8.0), and 1× protease inhibitor cocktail. Chromatins were acquired under ice-cold conditions, followed by sonication to obtain soluble sheared chromatin with an average DNA length of 200-500 bp. A 20 μL portion of chromatin was stored at -20 °C for the purpose of input DNA, while 100 μL of chromatin was designated for immunoprecipitation utilizing the anti-H3K18la antibody (PTM-1406, PTM Bio). The immunoprecipitation reactions were conducted using 10 μg of antibody, with overnight incubation at 4 °C. On the subsequent day, the samples were supplemented with 30 μL of protein beads and further incubated for 3 hours. Then, a wash was performed using 2 mM EDTA, 50 mM NaCl, 20 mM Tris/HCl (pH 8.1), 0.1% SDS, and 1% Triton X-100. This was followed by two washes with 1% deoxycholic acid, 1% NP-40, 250 mM LiCl, 10 mM Tris/HCl (pH 8.1), and 1 mM EDTA. Finally, the beads were washed twice with TE buffer at a 1× concentration, consisting of 1 mM EDTA and 10 mM Tris-Cl (pH 7.5). The material that was bound to the beads was subsequently eluted using 300 μL of an elution buffer containing 1% SDS and 100 mM NaHCO3. It was then subjected to treatment with RNase A at a final concentration of 8 μg/mL for a duration of 6 hours at 65 °C, followed by treatment with proteinase K at a final concentration of 345 μg/mL overnight at 45 °C. The process commenced with the utilization of immunoprecipitated DNA to establish sequencing libraries, in accordance with the guidelines outlined by the I NEXTFLEX® ChIP-Seq Library Prep Kit for Illumina® Sequencing (NOVA-5143-02, Bioo Scientific). Finally, the libraries were sequenced on an Illumina Novaseq 6000 platform using the PE 150 method.

### Data analysis

To ensure the quality of our reads, we employed Trimmomatic (version 0.36), which effectively filtered out low-quality reads 25. Subsequently, the remaining clean reads were aligned to the genome using Bwa (version 0.7.15) with a q value of 0.05 26. Samtools (version 1.3.1) was employed to eliminate any potential PCR duplicates 27. MACS2 software (version 2.1.1.20160309) was employed to identify peaks utilizing the default parameters, including a bandwidth of 300 bp, a model fold range of 5 to 50, and a q-value threshold of 0.05. To assign peaks to their corresponding genes, we determined the midpoint of each peak and assigned it to the gene whose TSS (Transcription Start Site) was closest to this midpoint 28. Furthermore, we utilized HOMER (version 3) to predict motif occurrence within the identified peaks. This analysis was conducted with default settings, considering a maximum motif length of 12 base pairs 29. To gain deeper insights into the biological functions and pathways associated with our findings, we employed ClusterProfiler (http://www.bioconductor.org/packages/release/ bioc/html/clusterProfiler.html) in the R package for GO (Gene Ontology, http://geneontology.org/) and KEGG (Kyoto Encyclopedia of Genes and Genomes, http://www.genome.jp/kegg/) enrichment analysis. The GO and KEGG enrichment analyses were computed through the application of a hypergeometric distribution, employing q-value threshold of 0.05 as a criterion.

### Statistical analysis

Results are presented as the mean ± SD and data analysis was conducted using GraphPad Prism (version 9.0, GraphPad Software Inc.). Statistical comparison between groups was performed using the student's t-test. For multiple group comparisons, the one-way analysis of variance (ANOVA) was employed. A significance level of *P* < 0.05 was considered statistically significant.

## Results

### Lactate promotes immune evasion by limiting tumor-infiltrating CD8^+^ T cells

**Figure 1:**
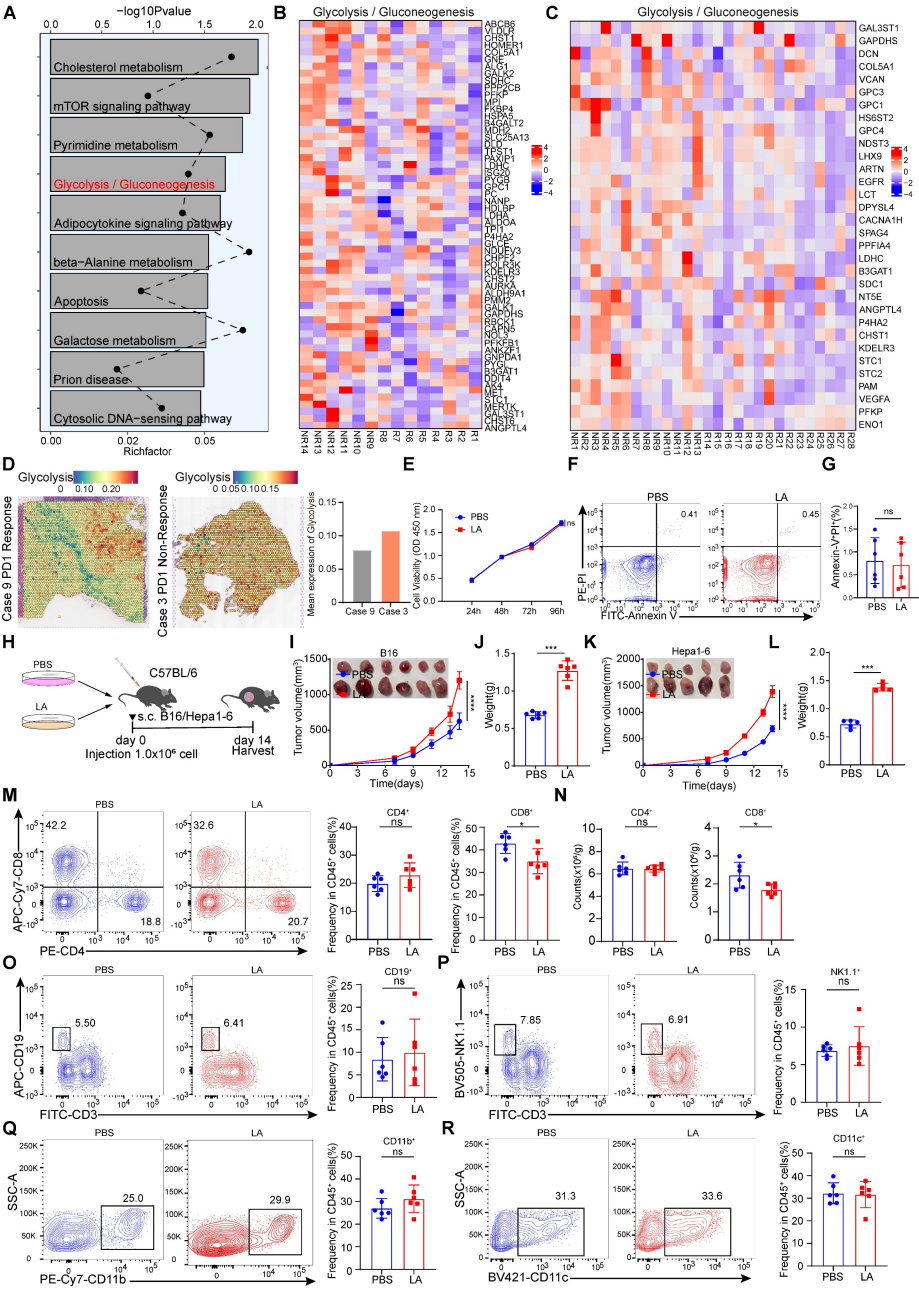
** Lactate inhibits anti-tumor immunity through CD8^+^ T cells. A** Kyoto Encyclopedia of Genes and Genomes (KEGG) analysis on RNA-sequencing (RNA-seq) data from melanoma patients who did not respond and those who responded to PD-1 treatment**. B** Heatmap of glycolysis-related genes expression in patients who did not respond and those who responded to PD-1 treatment (R: response, n = 8), (NR: non-response, n = 6)**. C** Heatmap of glycolysis-related genes expression in patients who did not respond and those who responded to PD-1 treatment (R: response, n = 15), (NR: non-response, n = 13)**. D** Spatial feature plots of signature score of glycolysis pathway in tissue sections.** E** The effects of L-lactate (LA, 20 mM) on B16 viability were assessed through utilization of the CCK-8 assay. **F-G** Annexin-V/PI staining was used to detect the effect of LA (20 mM) treatment on apoptosis by flow cytometry (n = 6). **H** Schematic representation of B16/Hepa1-6 cells (1×10^6^ cells/mouse) pretreated with PBS or LA (20 mM) for 3 days in C57BL/6 mice. **I-J** B16 tumor size, tumor growth curve and tumor weight were assessed (n = 6). Tumor volumes were determined through the application of the formula: 0.5 × (small diameter)^2^ × (large diameter). **K-L** Hepa1-6 tumor size, tumor growth curve and tumor weight were assessed (n = 5). Tumor volumes were determined through the application of the formula: 0.5 × (small diameter)^2^ × (large diameter). **M-N** Proportion and numbers of tumor-infiltrating CD8^+^ T and CD4^+^ T lymphocytes of tumor-bearing mice at 14 days by flow cytometry (n = 6). **O-R** The proportion of CD45^+^CD3^-^CD19^+^ B cells (O), CD45^+^CD3^-^NK1.1^+^ cells (P), CD45^+^CD11b^+^ myeloid cells (Q), CD45^+^CD11c^+^ DC (R) among CD45^+^ immune cells were quantified in B16 tumors via flow cytometry (n = 6). Data represent mean ± SD. Statistical significance was performed through the utilization of an unpaired t-test (G, J, L, M-R), two-way ANOVA (E, I, K), with the corresponding results expressed as follows: ns, non-significant, *p < 0.05, **p < 0.01, ***p < 0.001, ****p < 0.0001.

To investigate the key pathways that promote immune evasion, we conducted Kyoto Encyclopedia of Genes and Genomes (KEGG) analysis on RNA-sequencing (RNA-seq) data from melanoma patients who did not respond and those who responded to PD-1 treatment 30. The results showed that the glycolysis pathway was highly enriched in the non-responsive group to immunotherapy (Figure 1A). Subsequently, we analyzed the differentially expressed genes (DEGs) related to glycolysis in patients with and without response to immunotherapy and found that the expression of glycolysis-related genes was increased in non-responsive patients (Figure 1B-C). In addition, public data from spatial transcriptomic studies of hepatocellular carcinoma (HCC) revealed that the glycolysis pathway was highly enriched in non-responsive patients to immunotherapy (Figure 1D). Overall, these findings highlight the strong association between the glycolysis pathway and immune evasion. Metabolites of glycolysis, such as lactate, have been shown to be highly enriched in tumor tissue 31. To elucidate the intricate role of lactic acid in tumor biology, we conducted *in vitro* experiments using B16 cells. These cells were treated with L-lactate LA (20 mM) over a period of 3 days. CCK-8 assays were employed to evaluate cell proliferation, revealing that lactate made no difference to the viability of B16 cells (Figure 1E). Furthermore, flow cytometry analyses were employed to assess apoptosis rates. No significant alterations in apoptosis were observed in B16 cells following lactate treatment (Figure 1F-G). To further explore the role of lactate on anti-tumor immunity, lactate-pretreated B16/Hepa1-6 cells were used to establish a mouse tumor-bearing model (Figure 1H). Intriguingly, our results indicated that lactate treatment promoted tumor growth significantly in contrast to PBS treatment (Figure 1I-L). Determined to unravel the intricate TME governed by lactate-induced immunosuppression, we analyzed the proportion of multiple infiltrating immune cells. As expected, the lactate-pretreatment group showed remarkably reduced numbers of tumor-infiltrating CD8^+^ T cells in comparison with the control group, while the proportions of other immune cell subsets remained relatively unchanged (Figure 1M-R, Figure S1A). To investigate the role of lactate *in vivo*, we intraperitoneally injected C57BL/6 mice with sodium lactate, the basic form of lactate 32, before and after inoculation with B16/Hepa1-6 tumor cells (Figure S2A and F). The data showed that sodium lactate promoted tumor growth significantly in contrast to PBS treatment (Figure S2B-E, G-J). As expected, the lactate-treatment group showed remarkably reduced numbers of tumor-infiltrating CD8^+^ T cells in comparison with the control group, while the proportions of other immune cell subsets remained relatively unchanged (Figure S2K-T).

### Treatment with inhibition of lactate suppressed tumorigenesis

**Figure 2:**
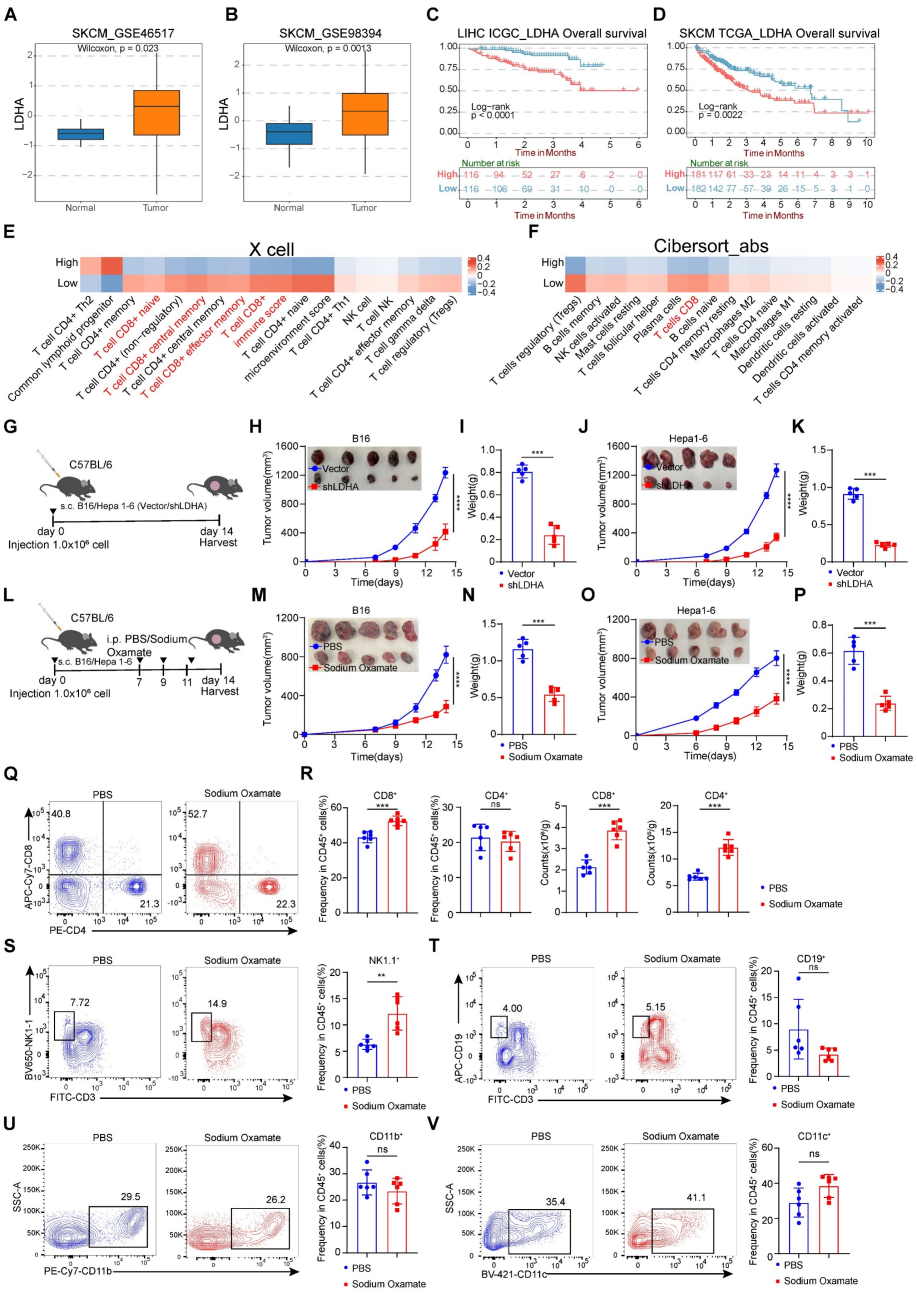
** Knockdown of LDHA or treatment with lactate dehydrogenase inhibitors inhibits tumorigenesis. A**-**B** The expression level of LDHA between melanoma and adjacent normal tissues. **C**-**D** Overall survival rates of hepatocellular carcinoma and melanoma from ICGC and TCGA database with high or low LDHA expression as defined by the median value. Statistical significance was determined by log-rank (Mantel-Cox) test. **E** Comparison of the scores for immune cells estimated by the xCell algorithm between high and low LDHA expression**. F** Comparison of the scores for immune cells estimated by the Cibesort abs algorithm between high and low LDHA expression**. G** Schematic representation of C57BL/6 mice subcutaneously injected with B16/Hepa1-6 Vector cells or B16/Hepa1-6 shLDHA cells.** H-I** B16 tumor volume, tumor growth curve (H) and tumor weight (I) were assessed between the control group (Vector) and shLDHA groups. Tumor volumes were determined through the application of the formula: 0.5 × (small diameter)^2^ × (large diameter) (n = 5).** J-K** Hepa1-6 tumor volume, tumor growth curve (J) and tumor weight (K) were assessed between the control group (Vector) and shLDHA groups. Tumor volumes were determined through the application of the formula: 0.5 × (small diameter)^2^ × (large diameter) (n = 5).** L** Schematic representation of C57BL/6 mice subcutaneously injected with B16 cells or Hepa1-6 cells and were treated with sodium oxamate (300 mg/kg).** M-N** B16 tumor volume, tumor growth curve (M) and tumor weight (N) were assessed between the control group and sodium oxamate treatment groups. Tumor volumes were determined through the application of the formula: 0.5 × (small diameter)^2^ × (large diameter) (n = 5).** O-P** Hepa1-6 tumor volume, tumor growth curve (O) and tumor weight (P) were assessed between the control group and sodium oxamate treatment groups. Tumor volumes were determined through the application of the formula: 0.5 × (small diameter)^2^ × (large diameter) (n = 5). **Q-R** Proportion and numbers of CD4^+^ and CD8^+^ T lymphocytes within the CD45^+^ immune cell in tumor-bearing mice (n = 6).** S-V** Quantification of CD45^+^CD3^-^NK1.1^+^ cells (S), CD45^+^CD3^-^CD19^+^B cells (T), CD45^+^CD11b^+^ myeloid cells (U), CD45^+^CD11c^+^ DC (V) among CD45^+^ cells by flow cytometry (n = 6). Data represent mean ± SD. Statistical significance was performed through the utilization of an unpaired t-test (A-B, I, K, N, P-V), log-rank (Mantel-Cox) test (C, D), two-way ANOVA (H, M, J, O), with the corresponding results expressed as follows: ns, non-significant, *p < 0.05, **p < 0.01, ***p < 0.001, ****p < 0.0001.

Tumor cells produce lactate through aerobic glycolysis, which exerts a significant inhibitory effect on CD8^+^ T cells in the tumor microenvironment. To investigate the clinical relevance of glycolysis in melanoma, we focused on the key enzyme LDHA in aerobic glycolysis and examined a series of expression and survival datasets from melanoma patients. We compared the expression levels of LDHA in melanoma and normal tissues and found that LDHA was significantly overexpressed in melanoma (Figure 2A-B). Subsequently, we conducted survival analysis and observed that higher LDHA transcript levels were associated with poorer overall survival (Figure 2C-D). Moreover, by using two independent algorithms, XCELL and CIBERSORT, to score the immune cell infiltration in melanoma patients, we found that patients with high LDHA expression had a negatively correlated level of CD8^+^ T cell infiltration (Figure 2E-F), suggesting a strong association between glycolytic product lactate and tumor immune evasion.

To further explore the connections between LDHA expression and CD8^+^ T cell-mediated anti-tumor effects, we constructed LDHA-Sh1 plasmids to knockdown LDHA (Figure 2G). Remarkably, LDHA knockdown significantly diminished B16 and Hepa1-6 tumor growth (Figure 2H-K). Next, we analyzed the characteristics of tumor-infiltrating lymphocytes and found the infiltration proportions of CD8^+^ T cells rather than other immune cell subsets were significantly increased (Figure S3A-J). We next evaluated whether lactate inhibition could attenuate tumorigenesis in melanoma and hepatocellular carcinoma. Herein, the LDH inhibitor (sodium oxamate) was adopted to attenuate lactate production. Tumor-bearing mice were systematically administered sodium oxamate (i.p.) at a dose of 300 mg/kg (Figure 2L). Remarkably, lactate inhibition significantly diminished B16 and Hepa1-6 tumor growth, as evidenced by the significantly decreased tumor weight in the sodium oxamate group contrasted with the control group (Figure 2M-P). Similarly, our analysis of tumor-infiltrating immune cells in B16 tumor revealed a noteworthy increase in the proportion of CD8^+^ T and NK cells in the sodium oxamate-treated group, while the proportions of other immune cell subsets showed no significant changes (Figure 2Q-V).

### Lactate increases B7-H3 expression in tumor cells by H3K18 lactylation

**Figure 3:**
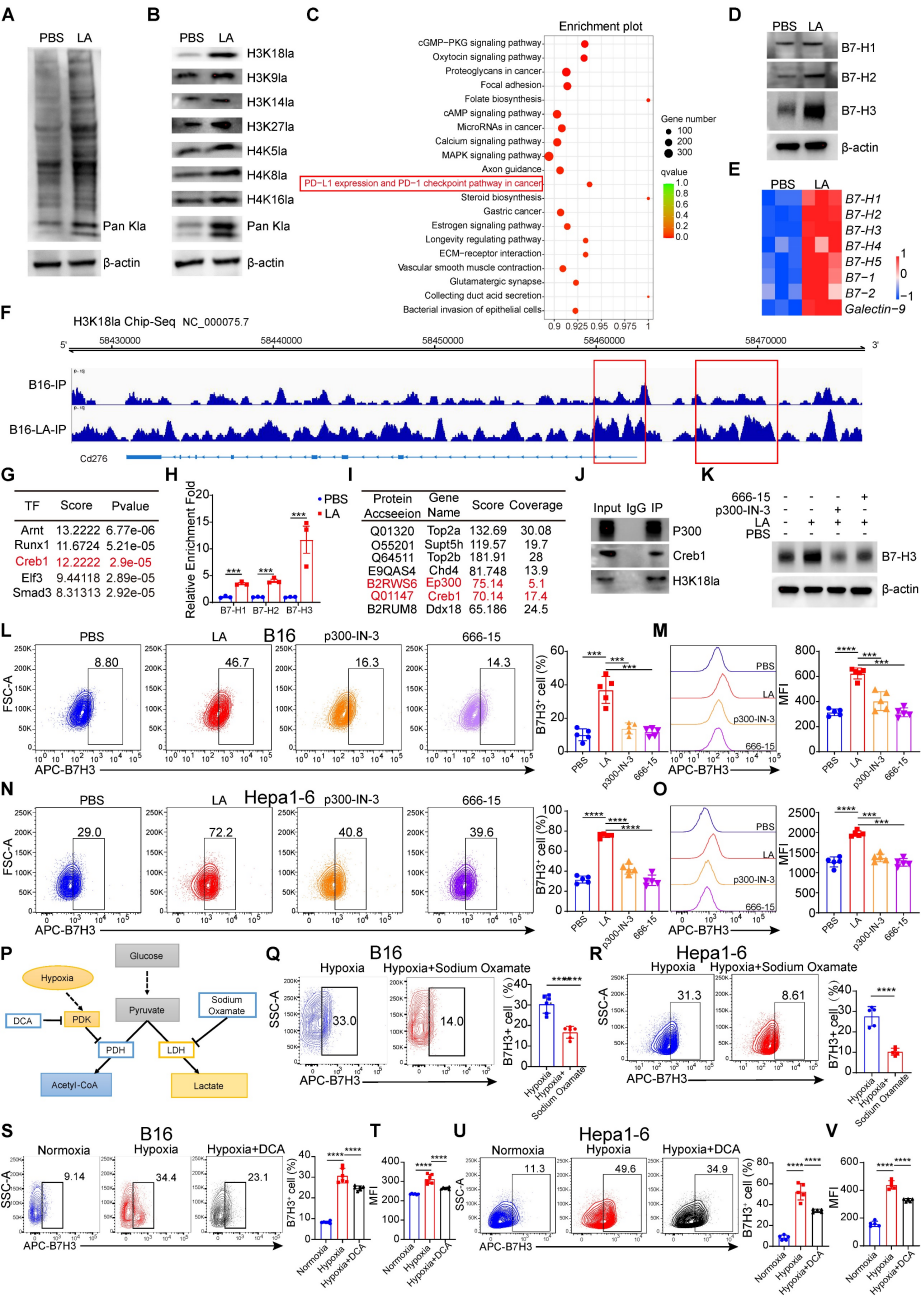
** H3K18 lactylation is responsible for the upregulation of B7-H3. A** Pan-lactylation was detected in B16 cells cultured in L-lactate (LA, 20 mM) for 3 days by Western blot.** B** Western blotting was used to analyze Site-specific histone lactylation levels and pan Kla in 10-15 kD from the whole-cell lysate of B16 cells subjected to a 20 mM LA treatment for 3 days**. C** Display plot of H3K18 la peak from Kyoto Encyclopedia of Genes and Genomes (KEGG) analysis. **D** Western blotting was used to analyze B7-H1, B7-H2 and B7-H3 expression levels from the whole-cell lysate of B16 cells subjected to a 20 mM LA treatment for 3 days**. E** Heatmap of mRNA expression of the inhibitory ligands, including B7-1, B7-H1, B7-H2, B7-H4, B7-H5, B7-1, B7-2 and Galectin-9.** F** IGV tracks for B7-H3 from H3K18la ChIP-seq analysis (n = 3). **G** Prediction of transcription factors for B7-H3 based on AnimalTFDB v4.0 and Jasper. **H** CUT-Tag qPCR results of B16 cells treated with PBS or 20 mM LA for 3 days by using anti-Creb1 immunoprecipitation. **I** Selected H3k18la binding proteins, protein score and sequence coverage in mass spectrometry are listed. **J** Immunoblot analysis of p300, Creb1 and H3k18la in cell lysates immunoprecipitated with anti-H3k18la or IgG from the whole-cell lysate of B16 cells subjected to a 20 mM LA treatment for 3 days. **K** Western blotting was used to analyze the expression level of B7-H3 in B16 cells treated with PBS, 20 mM LA, 1 μM p300-in-3 (a P300 inhibitor) + 20 mM LA and 1 μM 666-15 (a Creb1 inhibitor) +20 mM LA for 3 days. **L-M** Flow cytometry showed the Proportion and Mean Fluorescence intensity (MFI) of B7-H3 after being treated with PBS, 20-mM LA, 1 μM p300-in-3 + 20 mM LA and 1 μM 666-15 + 20 mM LA for 3 days in B16 cells (n = 5). **N-O** Flow cytometry showed the Proportion and Mean Fluorescence intensity (MFI) of B7-H3 after being treated with PBS, 20 mM LA, 1 μM p300-in-3 + 20 mM LA and 1 μM 666-15 +20 mM LA for 3 days in Hepa1-6 cells (n = 5). **P** Schematic representation of the regulation of lactate production by different metabolic modulators. **Q-U** Flow cytometric analysis was performed to evaluate the levels of B7-H3 expression in B16 cells and Hepa1-6 cells subjected to hypoxia (O_2_ < 1%) and treated either with or without sodium dichloroacetate (DCA, 20 mM) (S-V), (n = 6), as well as sodium oxamate (20 mM) (Q and R), (n = 6). Data represent mean ± SD. Statistical significance was conducted by means of unpaired t-test (H, Q, R) and one-way ANOVA comparisons test (L-O, S-V). ns, non-significant, *p < 0.05, **p < 0.01, ***p < 0.001, ****p < 0.0001.

Next, we investigated the mechanism underlying the impact of tumor cells affect CD8^+^ T cells through lactate. Given the stimulating effect of extracellular lactate on histone 16, consistent with our results, lactate treatment of B16 cells for 3 days increased the level of global lactylation (Figure 3A). Subsequently, we examined the currently known sites of histone lactylation modification. We found that the Pan-lysine lactylation (Pan Kla) and H3K18la exhibited the most significant changes in tumor cells treated with lactate (Figure 3B). Histone modification alterations can influence the transcriptional activation as well as repression of target genes 33. To reveal how histone lactylation affects tumor immunity by regulating gene expression, we employed Chromatin Immunoprecipitation sequencing (ChIP-seq) with anti-H3K18la antibodies. The KEGG analysis of the differentially up-regulated genes associated with H3K18la revealed enrichment in the PD-L1 expression and PD-1 checkpoint pathway in cancer (Figure 3C).

Subsequently, we demonstrated that lactate treatment significantly up-regulated the expression of B7-H1, B7-H2, and B7-H3 (Figure 3D). Notably, the increase in B7-H3 expression was the most pronounced among these markers. Consequently, our subsequent research will be directed towards elucidating the mechanisms by which H3K18la modulates the expression of B7-H3. Concurrently, we utilized qPCR to assess the impact of lactate on the expression of inhibitory ligands in tumor cells. The results corroborated that lactate promoted the expression of inhibitory ligands, with B7-H3 exhibiting the most significant change among them (Figure 3E). To ascertain whether histone lactylation could serve as a potential underlying cause for the upregulation of B7-H3 expression. Our subsequent investigations revealed a significant enrichment of H3K18la signal at the B7-H3 promoter region, which was further enhanced by lactate treatment (Figure 3F). Intriguingly, in the spacer region upstream of the promoter (Figure 3F), a region exhibited significantly increased enrichment after lactate treatment, indicating that may be a super-enhancer. It is plausible that lactate may promote gene transcription through positive feedback regulation of its promoter and super-enhancer formation 34, but the exact mechanism needs to be further verified. Therefore, utilizing the online tool AnimalTFDB v4.0 and Jasper, we predicted transcription factors that bind to the promoter region of B7-H3 based on its nucleotide sequence. We surprisingly found that the TFs that potentially bind to the promoter region of B7-H3 were including Arnt, Runx1, Creb1, Elf3 and Smad3 (Figure 3G). We conducted a retrospective analysis of the KEGG pathway results from the H3K18la ChIP-Seq data and identified the cGMP-PKG signaling pathway and cAMP signaling pathway as the top 10 pathways (Figure 3C). Creb1, a key transcription factor in both the cGMP-PKG signaling pathway and cAMP signaling pathway, may play a crucial role in the up-regulation of B7-H3 expression mediated by H3K18la through its activation (Figure 3G). CUT&Tag-qPCR results revealed a significant increase in Creb1 levels at the promoters of B7-H1, B7-H2, and B7-H3 in tumor cells following lactate stimulation, with the enrichment of Creb1 at the B7H3 promoter region being the most pronounced, suggesting a potential influence of Creb1 on the expression of B7-H3 (Figure 3H). To investigate potential epigenetic modifier interactions with H3K18la, we performed immunoprecipitation-mass spectrometry (IP-MS) on protein lysates from tumor cells using an H3K18la antibody. We identified Ep300, a known writer of lactylation modifications 35, as a potential binder to H3K18la (Figure 3I). More interestingly, it has been reported that Ep300 acts as a co-activator for the transcription factor Creb1 36. As expected, our immunoprecipitation data revealed that both Creb1 and Ep300 interact with H3K18la (Figure 3J). Subsequently, we treated tumor cells stimulated with lactate with inhibitors specific to Ep300 (Ep300-in-3) and Creb1 (666-15), and found that the induction of B7-H3 by lactate was declined both by Ep300 inhibitor and Creb1 inhibitor (Figure 3K). Public data mining also indicated a noteworthy positive correlation between Creb1-B7-H3 and Ep300-B7-H3 at the mRNA level (Figure S4A-D). The aforementioned results suggest that H3K18la may promote the expression of B7-H3 through the synergistic activation of Ep300 and Creb1, representing a potential underlying mechanism. Subsequently, we validated the findings using flow cytometry, which clearly demonstrated a significant up-regulation of B7-H3 expression at the protein level in the lactate-treated group compared to the control group (Figure 3L-O). Inhibitors of Ep300 and Creb1 notably suppressed the up-regulation of B7-H3 expression induced by lactate (Figure 3L-O).

In the cytoplasm, glucose is converted to pyruvate by a series of glycolytic enzymes. It is well known that hypoxia induces an elevation in lactate production 37. The inhibition of lactate production was achieved through the modulation of pyruvate dehydrogenase (PDK) and LDH activities by sodium dichloroacetate (DCA) and sodium oxamate, respectively (Figure 3P).

To evaluate the impact of inhibiting histone lactylation on B7-H3 expression, we employed small-molecule inhibitors of DCA and sodium oxamate to curtail lactate production and histone lactylation in B16 cells and Hepa1-6 cells. Hypoxia induces an increase in intracellular lactate production and histone Kla levels 38. Notably, exposure of B16 cells and Hepa1-6 cells to a hypoxic environment (O_2_ < 1%) for 48 hours resulted in increased B7-H3 expression compared to normal oxygen environment (Figure 3S-V). Given that sodium oxamate or DCA attenuate the induction of lactate production and histone Kla under hypoxic conditions, we administered these two lactate production inhibitors under hypoxic conditions. Encouragingly, the data demonstrated that the hypoxia-induced upregulation of B7-H3 expression was abrogated (Figure 3Q-V). Public data mining also indicated a noteworthy positive correlation between LDHA and B7-H3 at the mRNA level (Figure S5 A-B). Thus, our findings highlight the role of lactate in driving the upregulation of B7-H3 via histone lactylation, and offer evidence that inhibition of lactate production effectively curtails B7-H3 expression.

### Upregulation of tumor-expressed B7-H3 inhibits CD8^+^ T cell-dependent anti-tumor immunity

**Figure 4:**
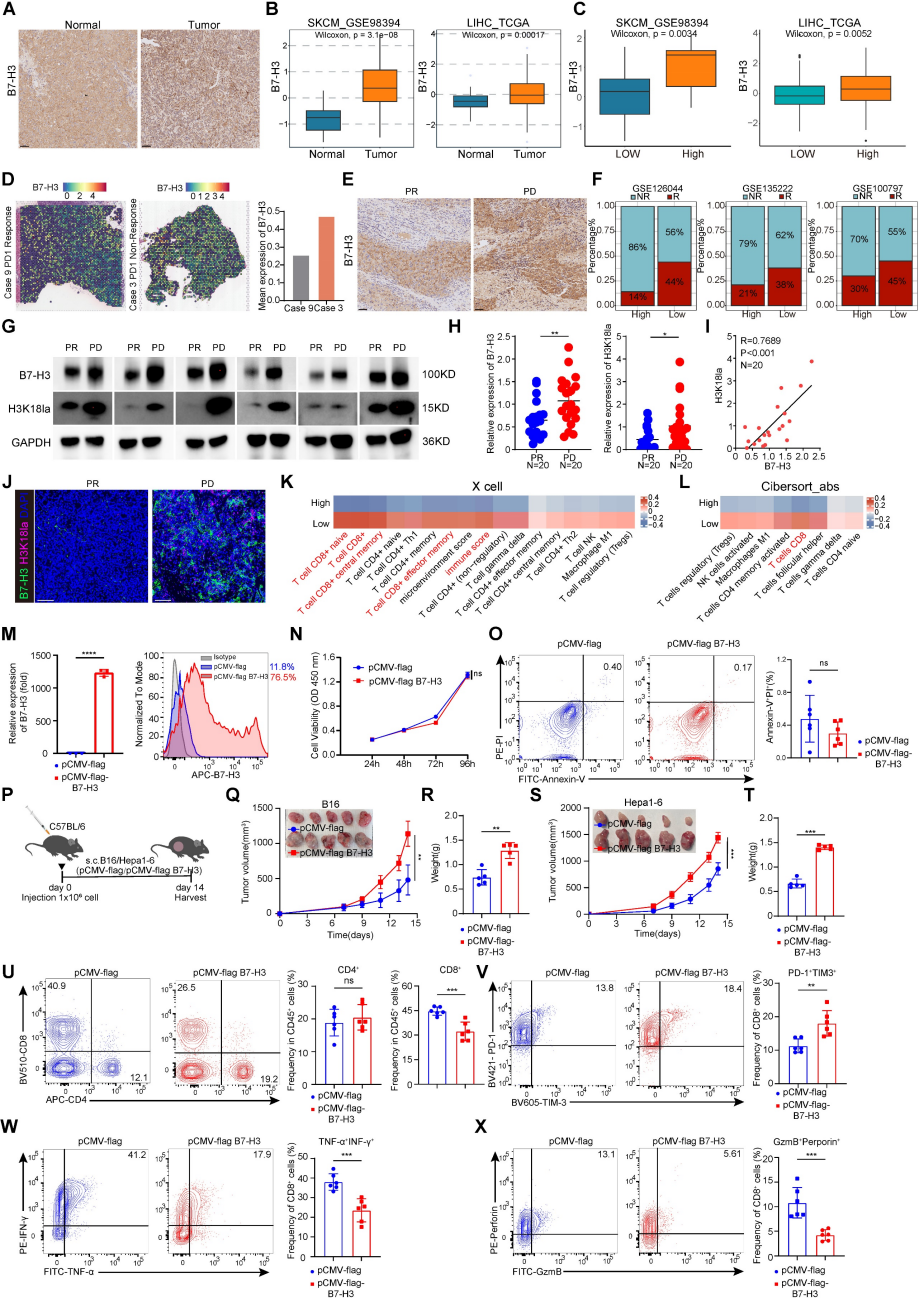
** B7-H3 overexpression reduces anti-tumor CD8^+^ T cell responses. A** Representative IHC images of B7-H3 staining of clinical HCC and paracancerous tissues. Scale bars, 50 μm. **B** The expression level of B7-H3 between tumor and adjacent normal tissue. **C** Results based on GSE98394 and TCGA database indicating the expression level of B7-H3 at different stages of SKCM and LIHC.** D** Spatial feature plots of signature score of B7-H3 in tissue sections. **E** The B7-H3 level in clinical HCC tissues that are responsive or non-responsive to PD-1 treatment. Scale bars, 50 μm. **F** Results based on GSE126044, GSE135222 and GSE100797 indicating the relationship between the high and low levels of B7-H3 expression and the response rate to immunotherapy.** G** Representative Western blot (WB) results showing the expression levels of B7-H3 and H3K18la from clinical HCC tissues with responsive or non-responsive to anti-PD-1 treatment (PR: Partial response; PD: Progressive disease). **H** The expression level of B7-H3 and H3K18la between PR (n = 20) and PD (n = 20) tumor tissues. **I** The correlation between H3K18la and B7-H3 in tumor tissues from PD patients (n = 20). **J** Immunofluorescent staining of clinical HCC tissues with responsive or non-responsive to anti-PD-1 treatment. B7-H3 (green), H3K18la (red), DAPI (blue). Scale bars, 50 μm. **K** Comparison of the scores for immune cells estimated by the xCell algorithm between high and low B7-H3 expression**. L** Comparison of the scores for immune cells estimated by the Cibesort abs algorithm between high and low B7-H3 expression**. M** B7-H3 overexpression as detected by q-PCR (n = 3) and flow cytometry. **N** CCK-8 assay was used to examine the effect of B7-H3 overexpression on cell viability. **O** The effect of B7-H3 overexpression on apoptosis of B16 cells was assessed via flow cytometry (n = 6).** P** Schematic diagram depicts C57BL/6 mice experimentally inoculated with B16/Hepa1-6 cells that have been transfected with empty vector (pCMV) or stable overexpressed B7-H3 (pCMV-B7-H3). **Q-R** B16 tumor Volume, tumor growth curve(Q) and tumor weight (R) were quantified. Tumor volumes were determined through the application of the formula: 0.5 × (small diameter)^2^ × (large diameter) (n = 5).** S-T** Hepa1-6 tumor Volume, tumor growth curve(S) and tumor weight (T) were quantified. Tumor volumes were determined through the application of the formula: 0.5 × (small diameter)^2^ × (large diameter) (n = 5). **U** The percentage of CD4^+^T and CD8^+^ T cells in pCMV and pCMV-B7-H3 Hepa1-6 tumors of the tumor-bearing mice at 14 days as determined by flow cytometry (n = 6).** V** The proportions of PD-1^+^TIM-3^+^subset within the CD8^+^ T cell was assessed in pCMV and pCMV-B7-H3 Hepa1-6 tumors (n = 6). **W** Quantification of TNF-α^+^ IFN-γ^+^ CD8^+^ T cells was conducted using flow cytometry in Hepa1-6 tumors of the tumor-bearing mice at day14 (n = 6).** X** The percentage of granzyme B (GZMB) and perforin co-expression in CD8^+^ T cells from Hepa1-6 tumors transfected with pCMV and pCMV-B7-H3 (n = 6). Data represent mean ± SD. Statistical significance was performed through the utilization of an unpaired t-test (H, M, O, R, T-X), two-way ANOVA (N, Q, S), purity-corrected Spearman test (I), with the corresponding results expressed as follows: ns, non-significant, *p < 0.05, **p < 0.01, ***p < 0.001, ****p < 0.0001.

We observed that B7-H3 expression was generally higher in tumor tissues compared to paracancerous liver tissues by using immunohistochemistry (Figure 4A). We compared the expression levels of B7-H3 between tumor and normal tissues, and found that B7-H3 was significantly highly expressed in tumor tissues (Figure 4B). We next compared the expression levels of B7-H3 between high-grade tumor cells and low-grade tumor cell, and found that B7-H3 was significantly overexpressed in high-grade tumor cells (Figure 4C). In addition, public data from spatial transcriptomic studies of HCC revealed that B7-H3 was highly expressed in non-responsive patients to immunotherapy (Figure 4D). By using immunohistochemistry in an HCC patient, we observed that B7-H3 expression was generally higher in non-responsive patients to immunotherapy (Figure 4E). Meanwhile, three datasets of anti-PD-1 immunotherapy revealed that B7-H3 was highly expressed in non-responsive patients to immunotherapy (Figure 4F). Subsequently, we collected tissue specimens from patients with liver cancer after immunotherapy. Western blot analysis revealed that in non-responders to immunotherapy, the levels of B7-H3 and H3K18la were significantly elevated compared to responders (Figure 4G-H). Moreover, there was a significant positive correlation between the protein levels of H3K18la and B7-H3 in non-responders (Figure 4I). Similarly, immunofluorescence results indicated a high enrichment of B7-H3 and H3K18la levels in non-responding patients after immunotherapy (Figure 4J). Moreover, by using two independent algorithms, XCELL and CIBERSORT, to score the immune cell infiltration in melanoma patients, we found that patients with high B7-H3 expression had a negatively correlated level of CD8^+^ T cell infiltration (Figure 4K-L), suggesting a strong association between B7-H3 and tumor immune evasion.

To research the function of B7-H3 in tumors, we constructed a B7-H3 overexpression vector, which was then transfected into B16 cells to establish a stable cell line overexpressing B7-H3. The qPCR and flow cytometry analysis verified that B7-H3 was successfully overexpression in B16 cells (Figure 4M). To assess whether B7-H3 overexpression could modulate cell proliferation and apoptosis, we conducted CCK-8 assays and Annexin V/PI staining. However, there were no significant changes in cell proliferation and apoptosis of B16 cells *in vitro* were observed between the transfection of the empty vector group and B7-H3 overexpression group (Figure 4N-O). These findings indicated that the B7-H3 of tumor cells did not act as a direct "tumor suppressor/oncogene".

As discussed previously, B7-H3 is a member of the B7 family of immune checkpoint proteins, provides a second signal for T cell activation 7. Given this, we assumed that B7-H3 may influence tumor cell elimination through its impact on tumor immunity, thereby influencing tumor progression and patient prognosis. Next, we injected B7-H3 overexpressing B16/Hepa1-6 cells into C57BL/6 mice via s.c. (Figure 4P).

Intriguingly, tumor growth in the B7-H3 overexpression group exhibited a significant acceleration in compared with the control (Figure 4Q-T). Subsequent flow cytometry analysis of tumor-infiltrating lymphocytes revealed a notable increase in the proportion of CD8^+^ T cells in the B7-H3 overexpression group, while CD4^+^ T cells displayed no significant alterations (Figure 4U). Compared with the control group, the proportion of CD8^+^ T cells expressing PD-1 and TIM-3 was increased in the B7-H3 overexpression group (Figure 4V and Figure S6A). Moreover, the ratio of cytotoxic cytokines TNF-α and IFN-γ secreted by CD8^+^ T cells was decreased in the B7-H3 overexpression group compared to the control group (Figure 4W and Figure S6A) Furthermore, the percentages of granzyme B^+^perforin^+^CD8^+^ T cells were decreased in the instances with B7-H3 overexpression compared to controls (Figure 4X and Figure S6A).

### Depletion of tumor-expressed B7-H3 augments anti-tumor immunity through CD8^+^ T cells

**Figure 5:**
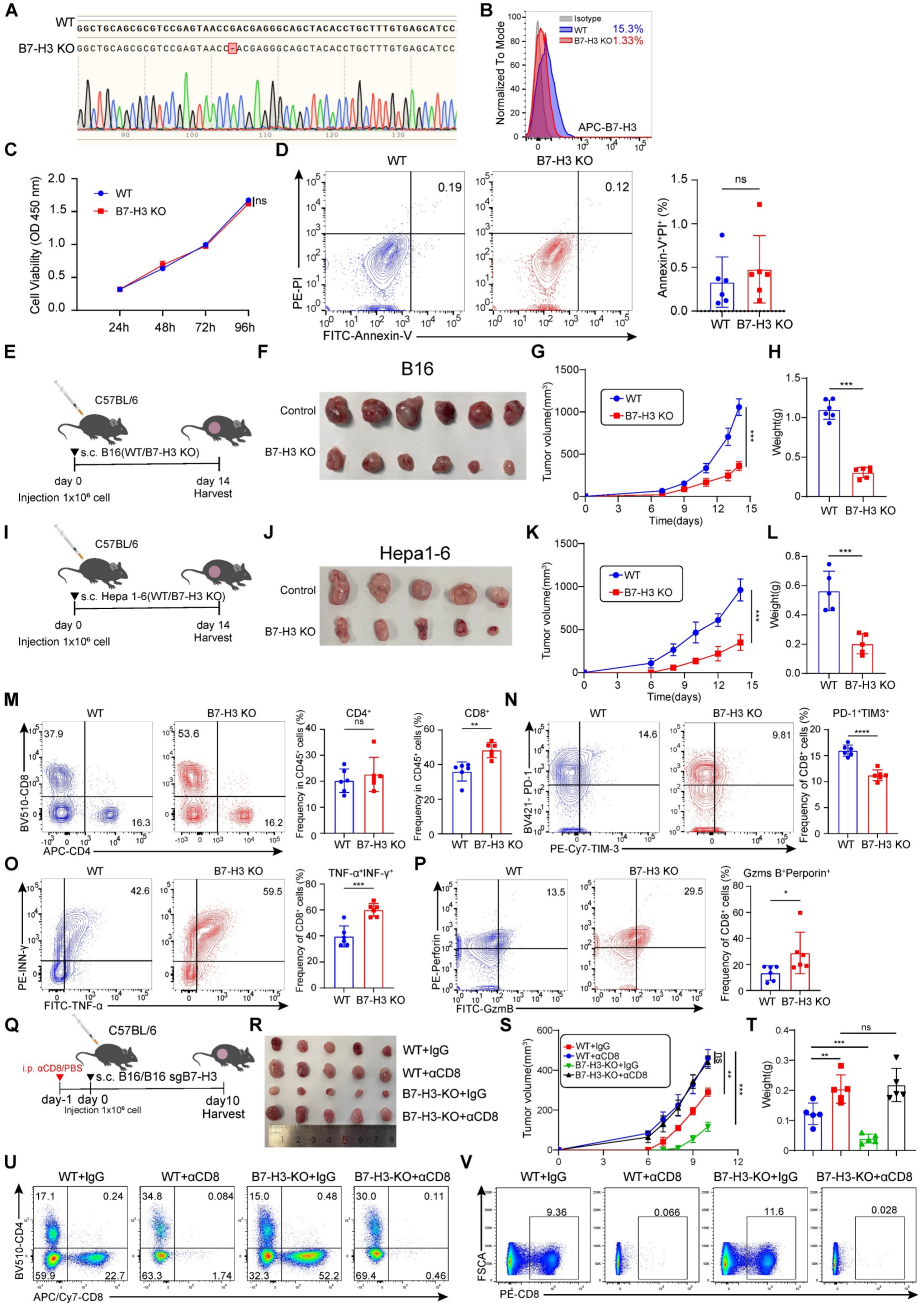
** B7-H3 deficiency in tumor cells augmented the CD8^+^ T cells anti-tumor immunity. A-B** The B7-H3 knockout B16 cell line was constructed based on CRISPR/Cas9. Validation of the effective knockout of B7-H3 within B16 cells was accomplished through sequencing (A) and flow cytometry (B).** C** CCK-8 was employed to ascertain the effect of B7-H3 knockout on the proliferation of B16 cells.** D** The effect of B7-H3 knockout on the apoptosis of B16 cells was detected utilizing flow cytometry (n = 6).** E** Schematic diagram illustrating the inoculation of C57BL/6 mice with either WT or B7-H3 KO B16 cells. **F**-**H** B16 tumor volume (F), tumor growth curve(G) and tumor weight (H) of B7H3 KO and B16 WT groups were assessed. Tumor volumes were determined through the application of the formula: 0.5 × (small diameter)^2^ × (large diameter) (n = 6).** I** Schematic diagram illustrating the inoculation of C57BL/6 mice with either WT or B7-H3 KO Hepa1-6 cells. **J**-**L** Hepa1-6 tumor volume (J), tumor growth curve(K) and tumor weight (L) of B7H3 KO and Hepa1-6 WT groups were assessed. Tumor volumes were determined through the application of the formula: 0.5 × (small diameter)^2^ × (large diameter) (n = 5).** M** The percentage of CD4^+^ and CD8^+^ T cells within the population of CD45^+^ cells on the day14 following B16 tumor inoculation was achieved through flow cytometry analysis (n = 6).** N** The Co-expression of PD-1 and TIM-3 in tumor-infiltrating CD8^+^ T cells was quantified in the murine tumor-bearing model among B16 KO group and B16 WT group. **O** Co-expression of IFN-γ and TNF-α in the proportion of tumor-infiltrating CD8^+^T cells in the B7-H3 KO group and B16 WT groups (n = 6).** P** The percentage of granzyme B^+^ (GZMB) perforin^+^ among tumor-infiltrating CD8^+^T cells between B7-H3 KO group and B16 WT groups (n = 6).** Q** Schematic diagram illustrating the inoculation of C57BL/6 mice with either WT or B7-H3 KO B16 cells treated with/without αCD8. **R**-**T** B16 tumor volume (R), tumor growth curve (S) and tumor weight (T) of WT + IgG, WT + αCD8, B7H3 KO + IgG and B7H3 KO + αCD8 groups were assessed. Tumor volumes were determined through the application of the formula: 0.5 × (small diameter)^2^ × (large diameter) (n = 5). **U-V** The quantification of CD8^+^ T cells within the population of CD45^+^ cells on the day10 following B16 tumor and spleen inoculation was achieved through flow cytometry analysis. Data represent mean ± SD. Statistical significance was performed through the utilization of an unpaired t-test (D, H, L, M-P), two-way ANOVA (C, G, K, S) and one-way ANOVA comparisons test (T) with the corresponding results expressed as follows: ns, non-significant, *p < 0.05, **p < 0.01, ***p < 0.001, ****p < 0.0001.

To further study the functional significance of B7-H3 in tumor cells, we generated a B7-H3 knockout cell line (B7-H3 KO) based on B16 cells using CRISPR/Cas9 system (Figure 5A-B). Subsequently, we conducted extensive *in vitro* analyses to confirm the influence of B7-H3 depletion on melanoma cell proliferation and apoptosis. CCK8 assays revealed no significant alterations in the proliferation of B16 cells upon B7-H3 knockout, while flow cytometry-based assessment exhibited comparable levels of apoptosis between the B7-H3 KO cells and wild-type (WT) counterparts (Figure 5C-D).

To comprehensively assess the impact of B7-H3 on tumor development *in vivo*, we established a tumor-bearing mouse model by subcutaneous inoculation of WT and B7-H3 KO B16 or Hepa1-6 cells. Tumor size was meticulously measured every two days, and after a two-week period, the mice were humanely sacrificed, and tumor tissues were isolated for subsequent analyses (Figure 5E and I). Remarkably, the tumor growth of tumor-bearing mice inoculated subcutaneously with B7-H3 KO cells was markedly impeded compared to those inoculated with WT cells. Consistently, the tumor volume and weight were notably reduced in the B7-H3 KO group (Figure 5F-H and J-L). T lymphocytes play a pivotal role as key effector cells in mounting effective anti-tumor immune responses. Previous studies have reported that inhibiting B7-H3 enhances the recruitment and cytotoxicity of T cells 38. We investigated whether B7-H3 knockout could enhance the infiltration and efficacy of effector CD8^+^ T lymphocytes in the TME. Our findings demonstrated a significant increase in the proportion of CD8^+^ T cells upon B7-H3 KO, while the proportion of tumor-infiltrating CD4^+^ T cells remained relatively unchanged between the groups (Figure 5M). Additionally, B7-H3 KO led to enhance the ability of CD8^+^ T cells to secrete IFN-γ and TNF-α (Figure 5O). To gain further insights into their cytotoxic capacity, we examined the expression of Granzyme B and perforin, revealing an elevated proportion of double-positive Granzyme B and perforin cells in B7-H3 KO B16 tumors compared to WT B16 tumors (Figure 5P). Furthermore, we assessed the expression of immune checkpoint receptors on tumor-infiltrating CD8^+^ T cells and observed a decreasing proportion of PD-1^+^TIM-3^+^CD8^+^ T cells within B7-H3 KO tumors in comparison to WT B16 tumors (Figure 5N). To further conform the function of CD8^+^ T cells, we depleted CD8^+^ T cells using anti-CD8 antibodies (Figure 5Q). Our data showed that depletion of CD8^+^ T cells reversed the anti-tumor effect and inhibited anti-tumor immunity in B7-H3 KO B16 tumors (Figure 5R-T). CD8^+^ T portions of tumor and spleen lymphocytes with or without anti-CD8 treatment were measured by flow cytometry (Figure 5U-V). Together, these findings provide compelling evidence that B7-H3 deficiency retards tumor progression by augmenting the CD8^+^ T cell-mediated anti-tumor immune response.

### Lactate inhibits anti-tumor immunity through B7-H3

**Figure 6:**
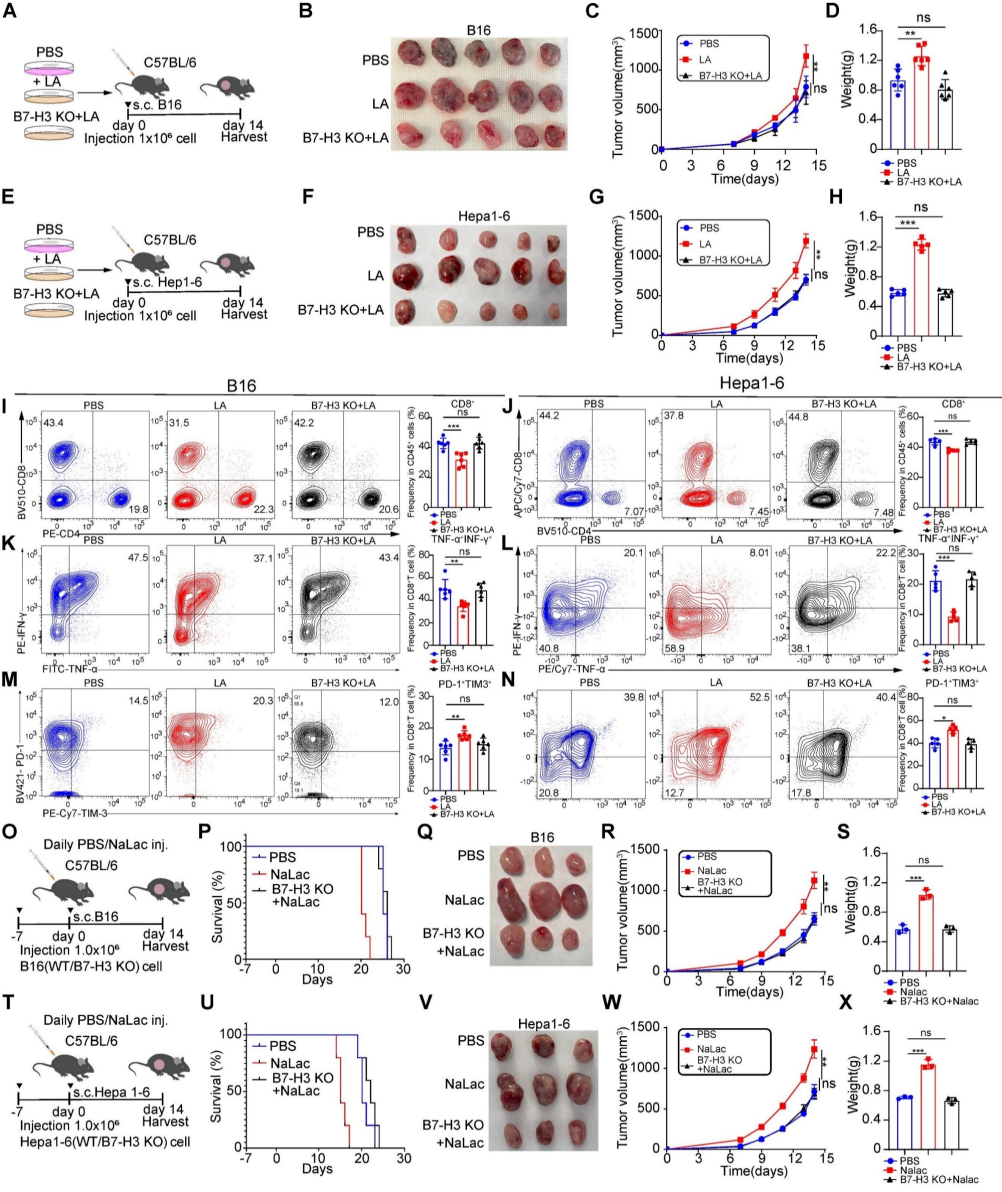
** The cancer-promoting effect of lactate is dependent on the presence of B7-H3. A and E** Schematic representation depicting the experimental design wherein C57BL/6 mice were injected with either WT or B7-H3 KO B16/Hepa1-6 cells that were pre-treated with or without L-lactate (LA, 20 mM) treatment. **B**-**D** The subsequent assessment of B16 tumor volume (B), tumor growth curve (C), and tumor weight (D) was conducted. The calculation of tumor volumes followed the formula: 0.5 × (small diameter)^2^ × (large diameter) (n = 5).** F-H** The subsequent assessment of Hepa1-6 tumor volume (F), tumor growth curve (G), and tumor weight (H) was conducted. The calculation of tumor volumes followed the formula: 0.5 × (small diameter)^2^ × (large diameter) (n = 5). **I and J** The percentage of CD8^+^ T cells within the subset of CD45^+^ immune cells present in tumor tissues derived from B16 tumor-bearing mice (n = 6) and Hepa1-6 tumor-bearing mice (n = 5).** K and L** Flow cytometry analysis was employed to calculate the ratios of IFN-γ^+^ TNF-α^+^ cells within the CD8^+^ cell population in tumor tissues derived from B16 tumor-bearing mice (n = 6) and Hepa1-6 tumor-bearing mice (n = 5).** M and N** The proportion of PD-1^+^TIM-3^+^ CD8^+^ T cells was employed to detect using flow cytometry in tumor tissues derived from B16 tumor-bearing mice (n = 6) and Hepa1-6 tumor-bearing mice (n = 5).** O and T** C57BL/6 mice were inoculated with either WT or B7-H3 KO B16/Hepa1-6 cells, and daily doses of mock (PBS) or 1 g kg^-1^ sodium lactate were administrated intraperitoneally. **P and U** Survival curves of B16/Hepa1-6 tumor-bearing C57 mice treated as indicated (n = 5). **Q-S** The subsequent assessment of B16 tumor volume (Q), tumor growth curve (R) and tumor weight (S) was conducted. Tumor volumes were determined through the application of the formula: 0.5 × (small diameter)^2^ × (large diameter) (n = 3). **V-X** The subsequent assessment of Hepa1-6 tumor volume (V), tumor growth curve (W) and tumor weight (X) was conducted. Tumor volumes were determined through the application of the formula: 0.5 × (small diameter)^2^ × (large diameter) (n = 3). Data represent mean ± SD. Statistical significance was performed through the utilization of two-way ANOVA comparisons test (C, G, Q, V), Log-rank (Mantel-Cox) test (P, U) and one-way ANOVA comparisons test (D, H, I-N, S, X), with the corresponding results expressed as follows: ns, non-significant, *p < 0.05, **p < 0.01, ***p < 0.001, ****p < 0.0001.

We found that lactate had the ability to promote melanoma and hepatocellular carcinoma progression, with its effect being mediated through the transcriptional upregulation of B7-H3 via histone lactylation. To determine whether the impact of lactate was dependent on B7-H3, we pretreated B16/Hepa1-6 WT cells and B7-H3 KO B16/Hepa1-6 cells with LA for 3 days to construct tumor-bearing model (Figure 6A and E). As anticipated, we observed larger tumor sizes in the lactate-treated group compared with vehicle group, consistent with our previous results. However, the growth rate, size, and weight of tumors in B7-H3 KO cells exposed to lactate displayed no significant differences compared to the vehicle group, indicating the loss of lactate's effect (Figure 6B-D and Figure 6F-H). Collectively, these observations establish the requirement for B7-H3 in mediating the promotion of tumor progression exerted by lactate. Furthermore, we found that LA treatment inhibited the infiltration of CD8^+^ T cells compared to the vehicle group. Interestingly, in the B7-H3 KO group treated with LA, the proportion of tumor-infiltrating CD8^+^ T cells did not differ significantly when compared to compared to the vehicle group (Figure 6I-J). Additionally, in comparison to the vehicle group, the lactate-treated group exhibited reduced secretion of cytokines TNF-α and IFN-γ by CD8^+^ T cells, alongside an increase in the expression of inhibitory molecules such as PD-1 and TIM-3. Notably, the addition of LA after B7-H3 KO showed no noticeable difference in the expression of these molecules compared to the vehicle group (Figure 6K-N).

To investigate the role of lactate *in vivo*, we intraperitoneally injected C57BL/6 mice with sodium lactate, the basic form of lactate, before and after inoculation with B16/Hepa1-6 tumor cells (Figure 6O and T). As anticipated, we observed larger tumor sizes in the sodium lactate-treated group compared to the vehicle control group, which is consistent with our previous findings. However, in B7-H3 knockout cells exposed to sodium lactate, there was no significant difference in tumor growth rate, size, and weight compared to the vehicle control group, indicating that the effect of lactate had been lost (Figure 6P-S and Figure 6U-X).

### Depletion of tumor-expressed B7-H3 and sodium oxamate enhances the anti-tumor efficacy of anti-PD-1 therapy

**Figure 7:**
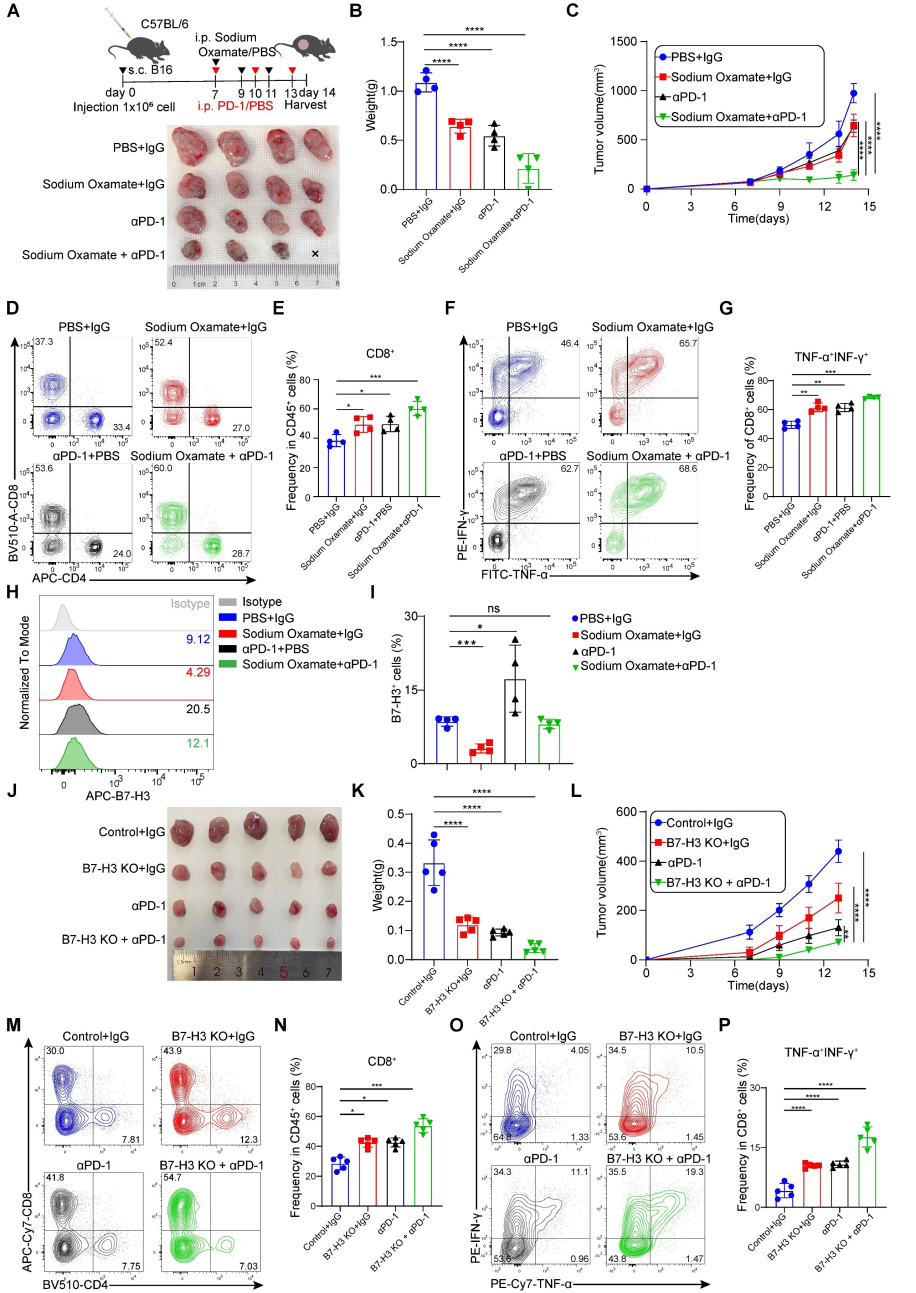
** Lactate dehydrogenase inhibitors combination with anti-PD-1 treatment accelerates tumor regression. A-C** Schematic diagram of C57BL/6 mice subcutaneously injected with B16 cells (1×10^6^ cell/mouse) were treated with sodium oxamate (300 mg/kg) anti-PD-1 (200 mg/mouse). Mice bearing B16 tumor were treated with sodium oxamate and anti-PD-1, and their combination (A). Tumor volume (A), tumor weight (B) and tumor growth curve (C) were assessed. Tumor volumes were determined through the application of the formula: 0.5 × (small diameter)^2^ × (large diameter) (n = 4). **D-E** Tumor tissues were isolated from tumor-bearing mice 14 days later, and subsequent flow cytometric analysis was performed to evaluate the percentages of tumor-infiltrating CD4^+^ T cells and CD8^+^ T cells (n = 4). **F-G** The co-expression of IFN-γ and TNF-α in CD8^+^ T cells was quantified in the indicated murine tumor model (n = 4). **H-I** Quantification of B7-H3 expression of tumor cells from tumor-bearing mice (n = 4). **J-L** Schematic diagram illustrating the inoculation of C57BL/6 mice with either control or B7-H3 KO Hepa1-6 cells treated with/without anti-PD-1. B16 tumor volume (J), tumor weight (K) and tumor growth curve (L) of Control + IgG, B7H3 KO + IgG, anti -PD-1, and B7H3 KO + anti-PD1 groups were assessed. Tumor volumes were determined through the application of the formula: 0.5 × (small diameter)^2^ × (large diameter) (n = 5). **M-N** Tumor tissues were isolated from tumor-bearing mice 13 days later, and subsequent flow cytometric analysis was performed to evaluate the percentages of tumor-infiltrating CD8^+^ T cells (n = 5). **O-P** The co-expression of IFN-γ and TNF-α in CD8^+^ T cells was quantified in the indicated murine tumor model (n = 5). Data represent mean ± SD. Statistical significance was conducted by means of one-way ANOVA comparisons test (B, E, G, I, K, N, P) and two-way ANOVA comparisons test (C, L), with the corresponding results expressed as follows: ns, non-significant, *p < 0.05, **p < 0.01, ***p < 0.001, ****p < 0.0001.

Studies have found that promote PD-1 expression in regulatory T (Treg) cells within high glycolysis tumor models. And PD-1 block therapy can activate PD-1^+^ Tregs, thereby suppressing PD-1^+^CD8^+^ T cells and ultimately leading to treatment failure 39. Therefore, reducing lactate levels in tumor cells may hold potential for improving the therapeutic efficacy of immunotherapies, such as PD-1 inhibitors. To explore this further, we evaluated the combination of LDH inhibitors with anti-PD-1 antibodies in terms of their therapeutic effects. In our experiments, mice bearing B16 cells were treated with sodium oxamate, anti-PD-1, or a combination of both, which revealed synergistic anti-tumor effects when sodium oxamate was combined with anti-PD-1 treatment compared to individual treatments alone (Figure 7A-C). This was accompanied by increased tumor infiltration CD8^+^ T cell, as well as elevated production of IFN-γ and TNF-α by CD8^+^ T cells (Figure 7D-G). Of note, we discovered that anti-PD-1 antibody therapy upregulated the expression of B7-H3, which could potentially contribute to treatment resistance (Figure 7H-I). Because of the synergistic effect of the two drugs in combination and the decreased expression of B7-H3 after sodium oxamate treatment, it is suggested that inhibition of lactate production may potentiate the anti-tumor efficacy of immunotherapy by down-regulating B7-H3. Moreover, B7-H3 ablation in tumors significantly enhanced PD-1 blockade efficacy and greatly delayed tumor growth (Figure 7J-L), and also dramatically restored anti-tumor immunity of CD8^+^ T cells (Figure 7M-N). Additionally, TNF-α^+^IFN-γ^+^CD8^+^T cells were dramatically increased in the combination group (Figure 7O-P). These results suggest that LDH inhibitors relieve lactate upregulation of B7-H3 mediated suppression of tumor-reactive CD8^+^ T cells, ultimately leading to tumor regression and synergistic effects with immune checkpoint blockade.

## Discussion

Although ICIs targeting PD-1 and CTLA-4 have proven to be effective in treating locally advanced or metastatic carcinoma, a significant portion of patients fail to respond to the treatment or experience early recurrence 40. The resistance mechanisms observed in immunotherapy, including neoantigen depletion 41, dysregulation of IFN-γ signaling 42, defects in antigen presentation 43, TME-mediated immunosuppression and rejection 44, 45, as well as the upregulation of other immune checkpoints besides PD-1 and CTLA-4 molecules 46, 47, potentially contribute to immune escape. Studies have revealed that ICIs therapy can lead to an upregulation of inhibitory receptors that inhibit T cell function 46, including VISTA and TIM-3 48. Interestingly, our research has demonstrated that anti-PD-1 treatment increases the expression of B7-H3 in tumor cells, which could be a factor in immune resistance. Previous studies have highlighted that the combination therapy of anti-PD-1 treatment and B7-H3 inhibition effectively inhibits tumor progression. Building upon this knowledge, our study has uncovered that lactate produced by tumor cells promotes the expression of B7-H3 in B16 cells. Intriguingly, investigations have shown that B16-F10 melanoma tumors deficient in LDHA exhibit increased infiltration of NK cells and CD8^+^ T cells. In addition, the administration of anti-PD-1 therapy has been shown to augment the anti-tumor immune response in melanoma-bearing mice generated from LDHA-deficient B16-F10 cells 49. Motivated by these findings, we explored the efficacy of combining LDH inhibitors with anti-PD-1 treatment. Reduction of lactate concentration, when combined with anti-PD-1 therapy, exhibits a more potent anti-tumor effect compared to anti-PD-1 treatment alone. Similarly, Gu *et al.* have discovered that lactate regulates the generation of Treg cells by catalyzing the lactylation of MOESIN protein and improving its interaction with the TGF-β receptor and downstream SMAD3 signaling 50. Importantly, the combined application of an LDH inhibitor and an anti-PD-1 antibody has been shown to synergistically enhance this anti-tumor effect 50.

In addition to targeting LDH, alternative strategies to regulate lactate production have emerged. One such approach involves the modulation of Monocarboxylate transporters (MCTs), responsible for lactate transportation. Inhibiting MCTs has been shown to increase intracellular lactate levels through the promotion of glycolysis. Qian *et al.* found that the knockdown of MCT4 inhibited the M2 polarization and abnormal T cell function induced by LKB1 deletion and reversed the resistance to PD-1 blockade 51. Furthermore, in a preclinical trial, the MCT1 inhibitor AZD3965 combined with anti-PD-1 antibody reduced the infiltration of exhausted PD-1^+^ Tim-3^+^ T cells and enhanced antitumor immunity in solid tumors 52. Therefore, targeting lactate in combination with immunotherapy represents a promising avenue for novel tumor therapies. Beyond elucidating the potential role of LDH inhibitors in cancer treatment, our study investigated the mechanism underlying lactate-mediated B7-H3 regulation.

The Warburg effect, an important characteristic of cancer energy metabolism, refers to the tendency of cancer cells to produce lactate through glycolysis even under the condition of sufficient oxygen 53. Conventionally, lactate has been considered a metabolic byproduct. However, recent studies have shown that lactate can mediate immunosuppression and play an important role in promoting tumorigenesis 54. Surprisingly, a single metabolite can have a powerful effect on immune cell function. Equally striking is the discovery by Zhang *et al.* of histone lactylation, a novel post-translational modification of proteins using lactate as a substrate, capable of regulating gene expression 55. Intriguingly, emerging studies have highlighted the involvement of histone lactylation in driving the progression of ocular melanoma 18, renal cancer 55, non-small cell lung cancer 17, colorectal cancer 19, 56, and other tumors, ae well as its role in facilitating tumor immune evasion 19, 57. The lactylation score in gastric cancer patients has negative correlation with immune cell infiltration and response to immune checkpoint inhibitors 58. H3K18la accelerates the development of melanoma by inducing the YTHDF2 gene 18. H3K18la also promotes trsncription of oncogene LCN2 and c-Myc, exerting a pro-tumor effect in breast cancer and bladder 59, 60. Notably, the tumor cells-derived lactate also induces PDL1 expression on neutrophil 61. Here, we found that in liver cancer patients, the level of H3K18la was significantly elevated in non-responders, compared to responders. Elevated H3K18la up-regulated B7-H3 expression and inhibit CD8^+^ T cell-mediated anti-tumor immune response. Moreover, anti-PD-1 treatment increased the expression of B7-H3 in tumor cells. All these indicated that H3K18la and B7-H3 may contribute to resistance to immunotherapy. The discovery of histone lactylation provides a blueprint for unraveling the mystery of the Warburg effect and for understanding the impact of lactate on tumor immunity 54, 57. Investigating the potential interplay between histone lactylation and tumor progression, as well as its regulatory effects on tumor gene expression, particularly within the context of the TME, holds great promise for future research endeavors.

Given that B7-H3 likely has a predominantly inhibitory role in anti-tumor immunity and may be a target in immunotherapy, clinical trials, including anti-B7-H3mAb only and combined with other approaches, have been initiated to determine the safety and efficacy of targeting B7-H3 in multiple cancer types 62. In phase I and II trial of enoblituzumab, there were no surgical delays or complications, meeting the study investigators' primary safety endpoint 62, 63, suggesting that B7-H3 mAb treatment as monotherapy or combined with PD-1 inhibitors is generally safe. B7-H3-directed antibody-drug conjugate (ADC) caused dose-limiting neutropenia and fatigue, but no dose-limiting toxicities were observed 64, 65. Moreover, autologous T lymphocyte chimeric antigen receptor (CAR-T) cells against B7-H3 have been initiated phase I and II trials in both solid tumor and hematologic cancers 66.

We found that lactate could upregulate B7-H3 expression in a histone lactylation-dependent manner. B7-H3 is overexpressed in most human cancers, and clinical trials of anti-B7-H3 antibodies against melanoma 67, head and neck squamous cell carcinoma 1, and non-small cell lung cancer are ongoing 43, but the underlying mechanisms of B7-H3 expression remains unclear. Notably, the work by Liu *et al.* demonstrated that mTORC1 can enhance B7-H3 expression to suppress antitumor T cell immunity 68. Expanding on these findings, our research demonstrated that lactate supplementation *in vitro* led to a significant increase in both transcriptional and protein levels of B7-H3. Additionally, we observed that hypoxic conditions, known to induce lactate production, resulted in elevated B7-H3 expression. Crucially, we observed that inhibiting lactate production using DCA and sodium oxamate attenuated B7-H3 expression. Although we demonstrated the role of Creb1 and Ep300 in regulating H3K18la and B7-H3 expression, we did not specifically inhibit H3K18la to clarify the role of H3K18la. This is one limitation of our study. Another limitation is that we did not perform clinical study using inhibitors of LDHA, H3K18la or B7-H3. in the future, specific drugs targeting H3K18la and anti-B7-H3 antibodies, combined with PDL-1 antibodies, may overcome immunotherapy resistance.

Recent years have witnessed a surge in research efforts dedicated to unraveling the intricacies of immunotherapy and the tumor metabolic microenvironment, resulting in a heightened understanding of these pathways and the emergence of novel integrated approaches to enhance tumor treatment 69, 70. While our study focuses primarily on lactate, it is crucial to acknowledge the critical roles played by other metabolites within the TME 71, 72. Future studies of modulating metabolism in combination with ICIs therapy are worthwhile. Approaches that combine metabolic targets with immunotherapy offer the greatest potential for improved clinical efficacy.

## Conclusions

To sum up, we found that lactate could upregulate B7-H3 expression of tumor cells through histone lactylation, thereby inhibiting the proportion and functional efficacy of tumor-infiltrating CD8^+^ T cells, ultimately facilitating immune evasion. Additionally, we observed that anti-PD-1 antibody treatment induces an elevation in B7-H3 expression, potentially contributing to the development of immunotherapy resistance. Notably, we observed a reduction in B7-H3 expression upon inhibition of lactate production. Furthermore, the combination of an LDH inhibitor and anti-PD-1 treatment demonstrated a synergistic effect, significantly impeding tumor progression. Collectively, these findings offer a novel perspective for clinically addressing tumors by targeting lactate metabolism alongside ICIs, furnishing a promising avenue for future therapeutic interventions.

## Supplementary Material

Supplementary figures and table.

## Abbreviations

ICIs: Immune checkpoint inhibitors
LDH: Lactate dehydrogenase
PD-1: Programmed cell death protein 1
PD-L1: Programmed cell death protein 1 ligand 1
CTLA4: Cytotoxic T lymphocyte antigen 4
TME: Tumor microenvironment
qPCR: Quantitative real-time PCR
ChIP-seq: Chromatin Immunoprecipitation sequencing
KEGG: Kyoto Encyclopedia of Genes and Genomes
GO: Gene Ontology
OS: Overall survival
PDK: Pyruvate dehydrogenase
DCA: Sodium dichloroacetate
Treg: Regulatory T cell
IFN-γ: Interferon-γ
MCTs: Monocarboxylate transporters
