# Supplementary material for: Histone lactylation-driven B7-H3 expression promotes tumor immune evasion
Source: Theranostics. 2025 Jan 13;15(6):2338–59. doi: 10.7150/thno.105947 (PMC11840737; doi:10.7150/thno.105947)
Supplement: Supplementary file 1 — Supplementary figures and table. [file thnov15p2338s1.pdf]

Supplementary Figures and Table:

A

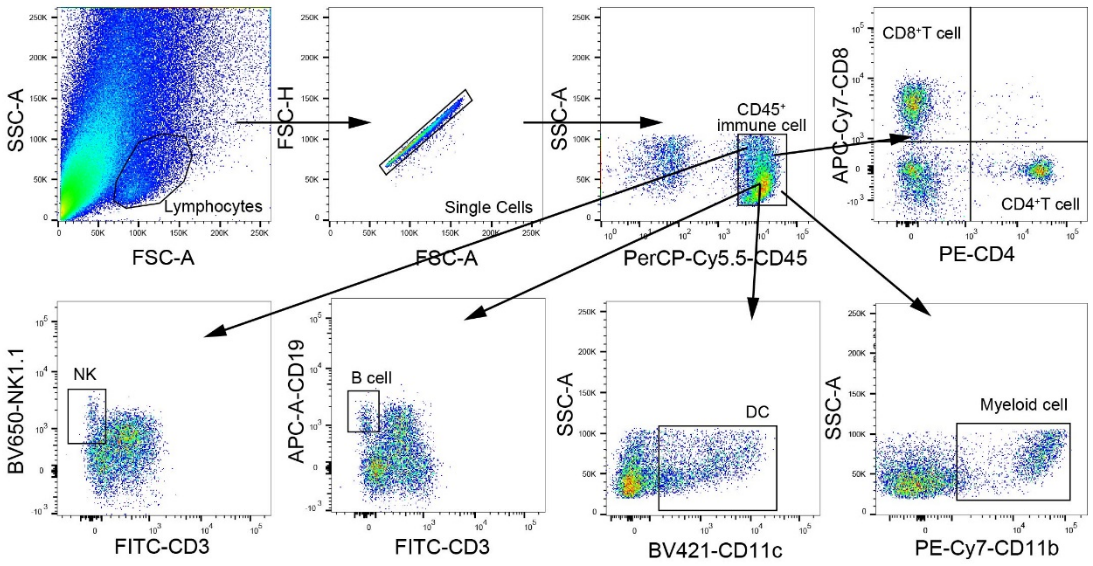

Figure S1: The gating strategy of multiple immune cells.

A The frequencies of tumor-infiltrating immune cells in tumor bearing mice were used by flow cytometry.

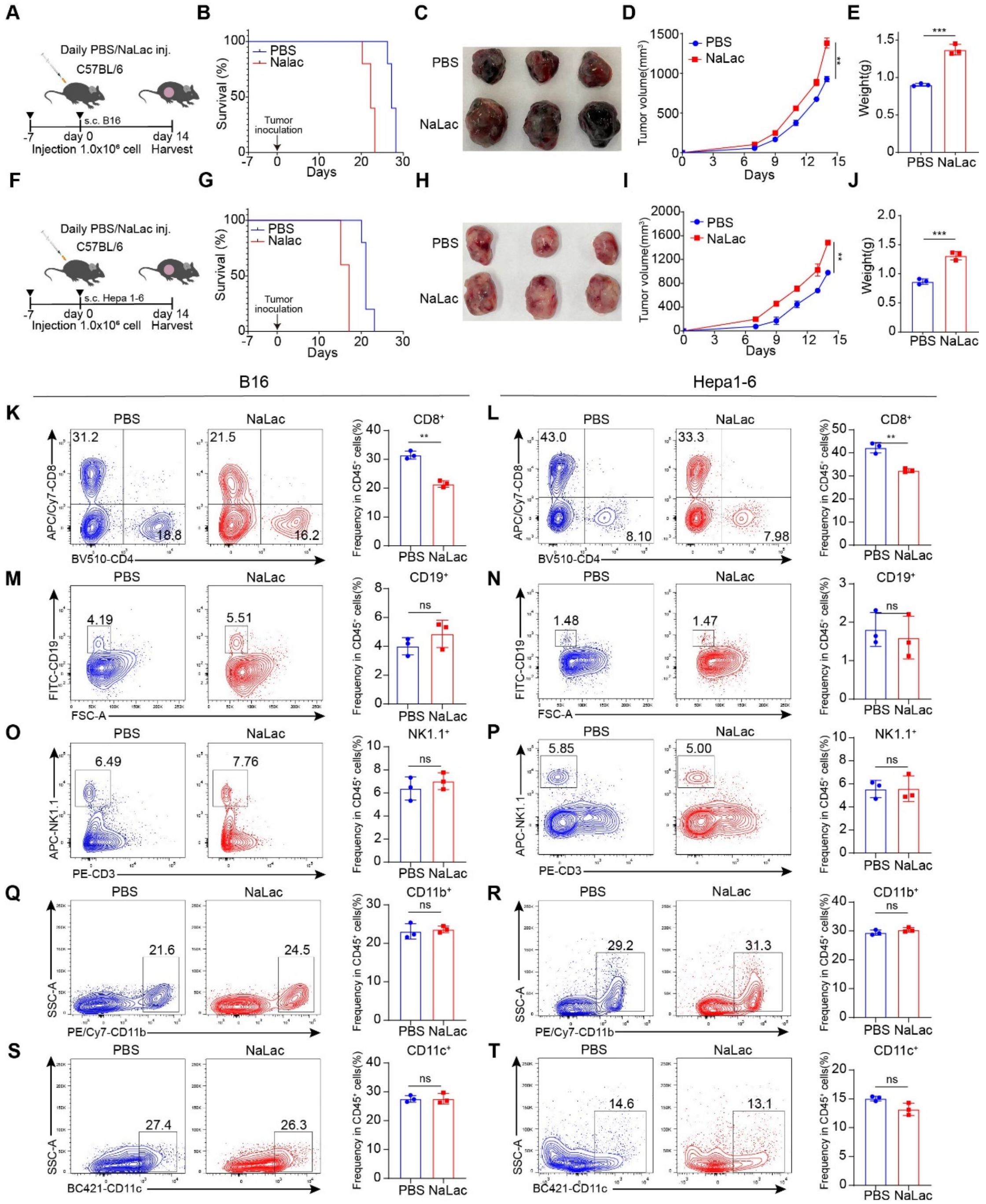

Figure S2: Lactate represses antitumor immunity through CD8$^+$ T cells, relative to fig1.

A C57BL/6 mice were inoculated with B16 tumor cells, and daily doses of mock (PBS) or 1 g kg^{-1} sodium lactate were administrated intraperitoneally. **B** Survival curves of B16 tumor-bearing C57 mice treated as indicated (n = 5). **C-E** B16 tumor volume (C), tumor growth curve (D) and tumor weight (E) were assessed between the control group (PBS) and Nalac treatment groups. Tumor volumes were determined through the application of the formula: 0.5 × (small diameter)^2 × (large diameter) (n = 3). **F**

C57BL/6 mice were inoculated with Hepa1-6 tumor cells, and daily doses of mock (PBS) or 1 g kg^{-1} sodium lactate were administrated intraperitoneally. **G** Survival curves of Hepa1-6 tumor-bearing C57 mice treated as indicated (n = 5). **H-J** Hepa1-6 tumor volume (H), tumor growth curve (I) and tumor weight (J) were assessed between the control group (PBS) and Nalac treatment groups. Tumor volumes were determined through the application of the formula: $0.5 \times (\text{small diameter})^2 \times (\text{large diameter})$ (n = 3). **K-L** Proportion of $CD8^+$ T lymphocytes within the $CD45^+$ immune cell in B16 (K)/Hepa1-6 (L)tumor-bearing mice (n = 3). **M-T** The proportion of $CD45^+CD3^-CD19^+$ B cells (M-N), $CD45^+CD3^-NK1.1^+$ cells (O-P), $CD45^+CD11b^+$ myeloid cells (Q-R), $CD45^+CD11c^+$ DC (S-T) among $CD45^+$ immune cells were quantified in B16/Hepa1-6 tumors via flow cytometry (n = 3). Data represent mean ± SD. Statistical significance was performed through the utilization of an unpaired t-test (E, J, K-T), two-way ANOVA (D, I) or Log-rank (Mantel–Cox) test was used for (B) and (G), with the corresponding results expressed as follows: ns, non-significant, *$p < 0.05$, **$p < 0.01$, ***$p < 0.001$, ****$p < 0.0001$.

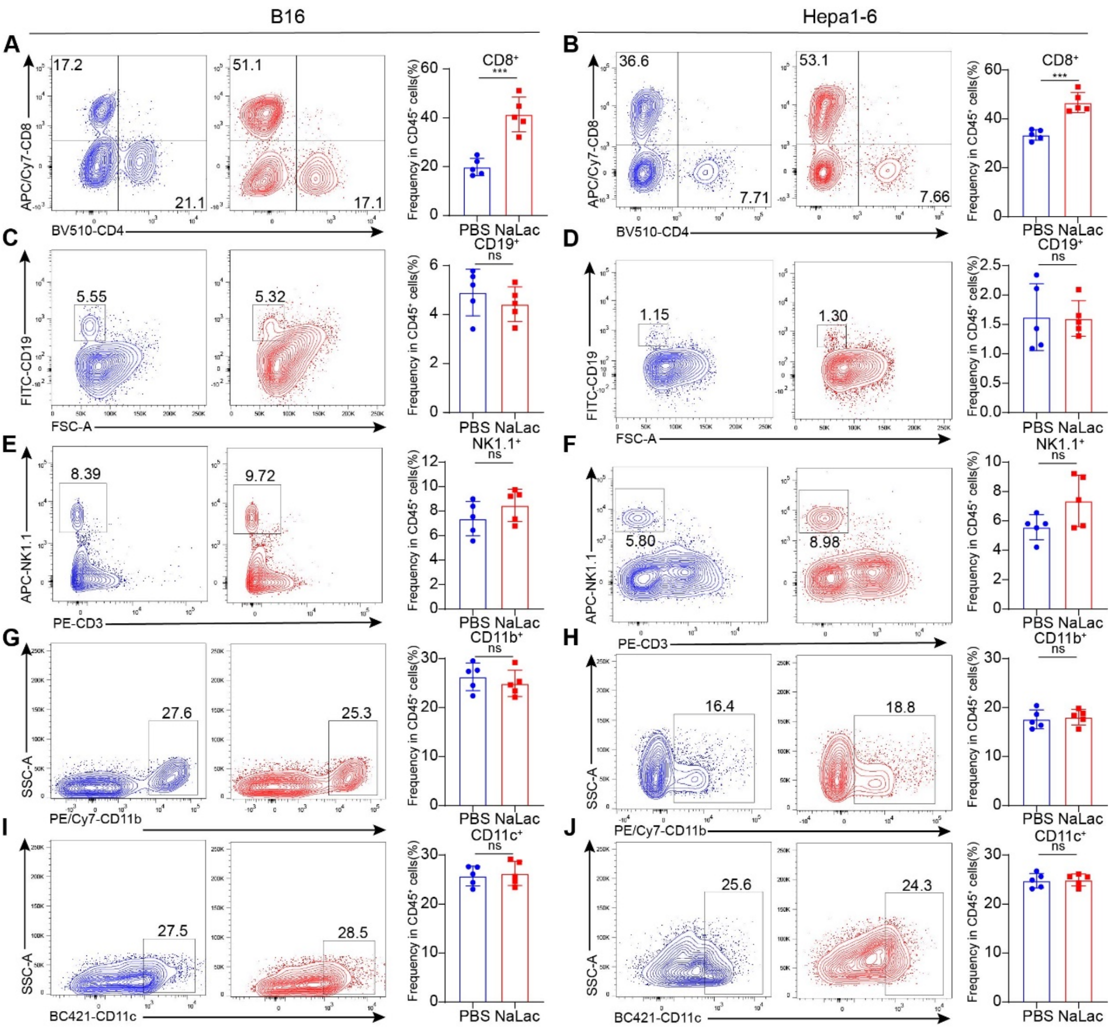

Figure S3: Knockdown of LDHA inhibits tumorigenesis through CD8$^+$ T cells, relative to fig 2.

A-B Proportion of CD8$^+$ T lymphocytes within the CD45$^+$ immune cell in B16/Hepa1-6 tumor-bearing mice (n = 5). **C-J** Quantification of CD45$^+$CD3$^-$CD19$^+$B cells (C-D), CD45$^+$CD3$^-$NK1.1$^+$ cells (E-F), CD45$^+$CD11b$^+$ myeloid cells (G-H), CD45$^+$CD11c$^+$ DC (L-J) among CD45$^+$ cells by flow cytometry (n = 5). Data represent mean ± SD. Statistical significance was performed through the utilization of an unpaired t-test (A-J), with the corresponding results expressed as follows: ns, non-significant, *$p < 0.05$, **$p < 0.01$, ***$p < 0.001$, ****$p < 0.0001$.

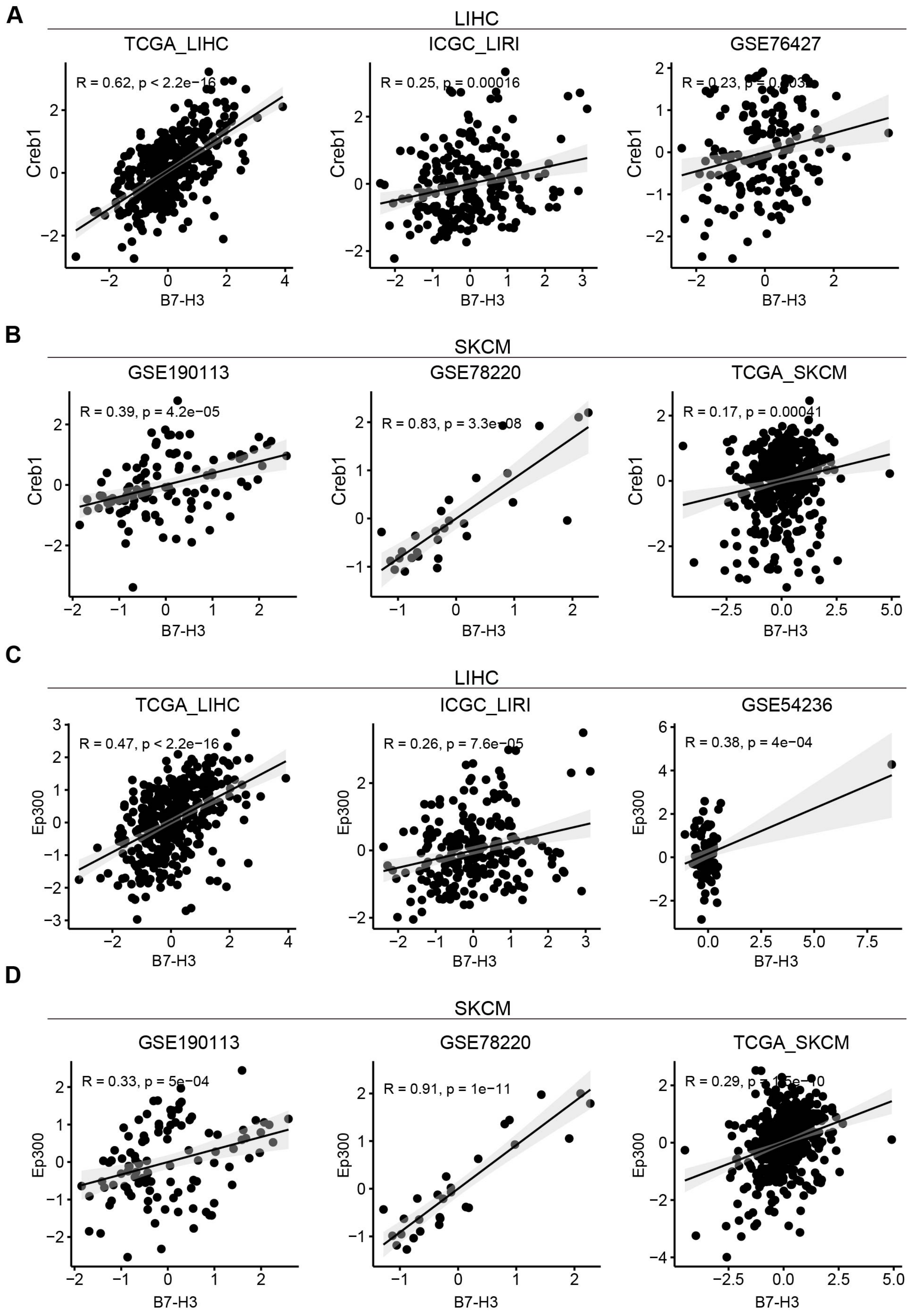

Figure S4: Public data were used to analyze the correlation between Ep300-B7-H3 and Creb1-B7-H3.

A The correlation between Creb1 and B7-H3 in public data from hepatocellular carcinoma patients. **B** The correlation between Creb1 and B7-H3 in public data from

melanoma patients. **C** The correlation between Ep300 and B7-H3 in public data from hepatocellular carcinoma patients. **D** The correlation between Ep300 and B7-H3 in public data from melanoma patients.

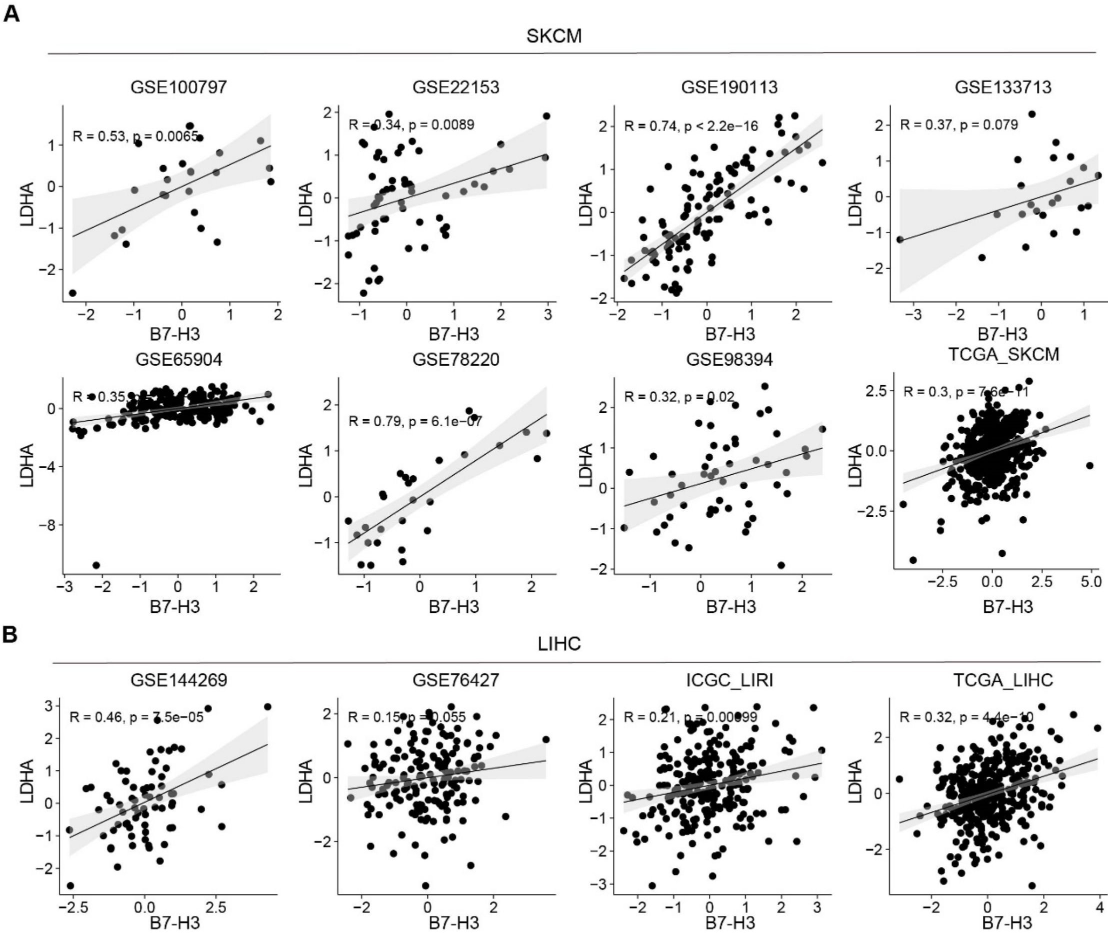

Figure S5: Public data were used to analyze the correlation between LDHA and B7-H3.

A The correlation between LDHA and B7-H3 in public data from melanoma hepatocellular carcinoma patients. **B** The correlation between Creb1 and B7-H3 in public data from hepatocellular carcinoma patients.

A

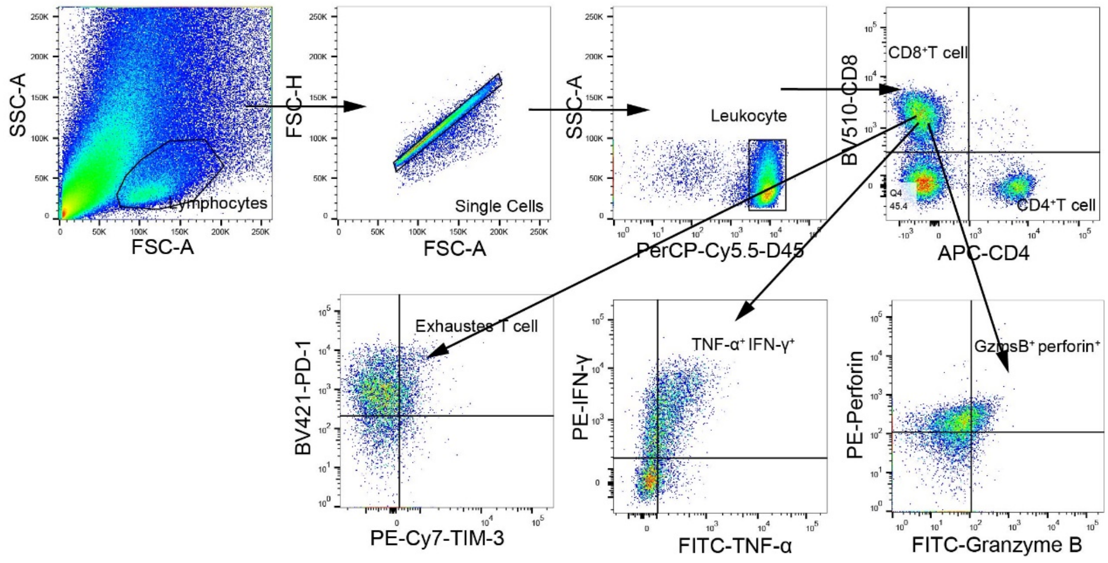

Figure S6: The gating strategy of CD8^{+}T cell functional phenotypes.

A Cell population analysis was gating from CD8, including exhausted T cells (PD-1^{+}TIM-3^{+}CD8^{+}), TNF-α^{+} IFN-γ^{+}CD8^{+}T cell and Granzyme B^{+} Perforin^{+}CD8^{+}T cell.

Table S1:

Antibodies	Source	Identifier
CPCy 5.5 anti-mouse CD45	Biolegend	103132
APC anti-mouse CD4	Biolegend	100412
BV510 anti-mouse CD4	Biolegend	300546
APC/Cy7 anti-mouse CD8	Biolegend	100713
BV510 anti-mouse CD8	BD	563068
FITC anti-mouse PD-1	Biolegend	135214
PE /Cy7 anti-mouse CD366	Biolegend	119716
FITC anti-mouse TNF-α	Biolegend	506304
PE anti-mouse IFN-γ	Biolegend	505808

BV650 anti-mouse NK1.1	Biolegend	108736
APC anti-mouse CD19	Biolegend	17-0193-82
BV421 anti-mouse CD11c	Biolegend	117343
PE-CY7 anti-mouse CD11b	Biolegend	101216
FITC anti-mouse CD3	Biolegend	100204
AF647 anti-mouse CD276	BD	562862
PE anti-mouse Perforin	Biolegend	154306
FITC anti-mouse,human GraznemyB	Biolegend	396404
BV605 anti-mouse CD366	Biolegend	119721
PE anti-mouse CD4	Biolegend	100408
APC/Cy7 anti-mouse CD8	Biolegend	100714
BV421 anti-mouse CD279	Biolegend	135221
